# Configuration of adaptable template RNA architectures to unfold the editable space of a nuclease prime editor

**DOI:** 10.1093/nar/gkaf522

**Published:** 2025-06-11

**Authors:** Pingbo Chen, Xiangyang Li, Qian Zhou, Jingzhou Chen, Lijin Lu, Pei Wang, Guiquan Zhang, Dongxiao Sun, Xingxu Huang, Jianghuai Liu, Xiaolong Wang

**Affiliations:** Hainan Institute of Northwest A&F University, Sanya 572025, China; International Joint Agriculture Research Center for Animal Bio-Breeding of Ministry of Agriculture and Rural Affairs, College of Animal Science and Technology, Northwest A&F University, Yangling, Shaanxi 712100, China; The Key Laboratory of Pancreatic Diseases of Zhejiang Province, The First Affiliated Hospital, Zhejiang University School of Medicine, Hangzhou 310003, China; Hainan Institute of Northwest A&F University, Sanya 572025, China; International Joint Agriculture Research Center for Animal Bio-Breeding of Ministry of Agriculture and Rural Affairs, College of Animal Science and Technology, Northwest A&F University, Yangling, Shaanxi 712100, China; Hainan Institute of Northwest A&F University, Sanya 572025, China; International Joint Agriculture Research Center for Animal Bio-Breeding of Ministry of Agriculture and Rural Affairs, College of Animal Science and Technology, Northwest A&F University, Yangling, Shaanxi 712100, China; Hainan Institute of Northwest A&F University, Sanya 572025, China; International Joint Agriculture Research Center for Animal Bio-Breeding of Ministry of Agriculture and Rural Affairs, College of Animal Science and Technology, Northwest A&F University, Yangling, Shaanxi 712100, China; State Key Laboratory of Pharmaceutical Biotechnology and MOE Key Laboratory of Model Animals for Disease Study, Model Animal Research Center at Medical School of Nanjing University, Nanjing 210061, China; The Second People's Hospital of Changzhou, the Third Affiliated Hospital of Nanjing Medical University, Changzhou 213000, China; Department of Animal Genetics and Breeding, College of Animal Science and Technology, Key Laboratory of Animal Genetics, Breeding and Reproduction of Ministry of Agriculture and Rural Affairs, National Engineering Laboratory for Animal Breeding, China Agricultural University, Beijing 100193, China; The Key Laboratory of Pancreatic Diseases of Zhejiang Province, The First Affiliated Hospital, Zhejiang University School of Medicine, Hangzhou 310003, China; School of Life Science and Technology, ShanghaiTech University, Shanghai 201210, China; State Key Laboratory of Pharmaceutical Biotechnology and MOE Key Laboratory of Model Animals for Disease Study, Model Animal Research Center at Medical School of Nanjing University, Nanjing 210061, China; Hainan Institute of Northwest A&F University, Sanya 572025, China; International Joint Agriculture Research Center for Animal Bio-Breeding of Ministry of Agriculture and Rural Affairs, College of Animal Science and Technology, Northwest A&F University, Yangling, Shaanxi 712100, China

## Abstract

The nuclease prime editor (PEn) combines double-strand break (DSB) induction with reverse transcription for editing. Recently, high-activity PEn forms (e.g. uPEn) have been developed via the concomitant application of DNA repair regulator(s). While the standard uPEn introduces edits only downstream of the nuclease-induced DNA break, we seek innovative designs to enable upstream-directed editing by re-configuring guide/template RNAs to drive prime edits into the target strand (TS), instead of the conventional non-TS. We first devise a dual-RNA uPEn strategy by supplementing a cleavage-competent sgRNA with an accessory template RNA for modifying target strand (ActRNA:t). Characterization of the dual-RNA system allows us to next develop a bifunctional target strand-programming pegRNA (tsp-pegRNA). Both the dual- and single-RNA upstream-modifying uPEn forms (versions 3.1/3.2) successfully drive diverse types of accurate edits into a panel of locations refractory to the standard uPEn and the latest nickase PE. Moreover, we provide insights on the role of uPEn's helper module (i.e. i53) in driving TS prime edits. Additional co-administration of a DNA-dependent protein kinase inhibitor with uPEn3.2 leads to further optimization of editing purities. Together, these advances transform uPEn into a highly applicable tool with much-expanded editable space, and lay a strong foundation for future development of PEn/PE platforms.

## Graphical Abstract

**Graphical Abstract:**
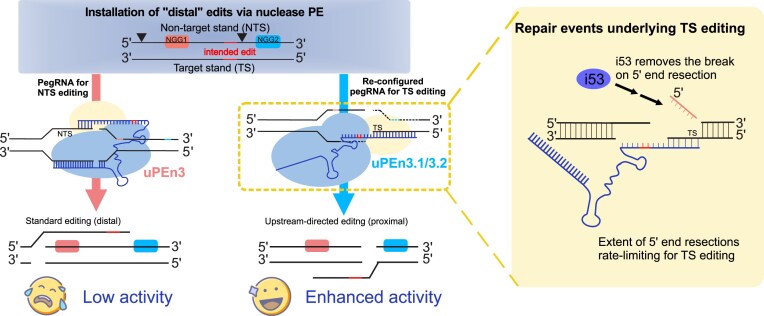

## Introduction

The advent of CRISPR/Cas [clustered regularly interspacedpalindromic repeats (CRISPR)/CRISPR-associated protein] genome editing technology has reshaped the landscape of genetic approaches in cells and organisms, and has indicated an exciting direction for therapeutically correcting pathogenic mutations [1, 2]. For a common genome editing practice, a programmed Cas9 nuclease is often adopted to induce a target-specific double strand DNA break (DSB), which subsequently proceeds through options of competing DNA repair pathways toward different editing outcomes. While the non-homologous end joining (NHEJ) process efficiently drives imprecise edits, homology-dependent repair (HDR) can compete with NHEJ and establish precise edits in accordance with the sequence of an introduced repair template [1, 2]. This initial progress has inspired continuous developments of more advanced tools toward precise genome editing [3, 4].

The recently developed prime editor (PE) represents a key addition to precise editing tools, capable of specifically installing various types of small-sized edits without the need for an intermediate DSB and a co-introduced DNA template [5]. The PE protein features a Cas9 nickase (H840A nCas9) module in fusion with a reverse transcriptase (RT), and employs a prime editing guide RNA (pegRNA). A pegRNA differs from a single-guide RNA (sgRNA) by an additional 3′-extension sequence that can be subdivided, in the 3′-to-5′ direction, into a primer-binding site (PBS) and an edit-encoding reverse transcription template (RTT). Such a design enables a pegRNA to serve dual roles as a targeting guide (with its sgRNA module) and as a reverse transcriptional template for the desired edit [with its 3′-extended module]. Upon recognizing the target sequence in the genome, a PE would first nick the non-targeted strand (NTS) to generate a 3′-OH end, and would subsequently reverse-transcribe the pegRNA-encoded template information into the NTS. This would then be followed by the actions of cellular DNA repair to complete the desirable modifications in both strands [5].

In extensive tests involving diverse cell types and systems, the PE has demonstrated impressive versatility and editing purity [3]. On the other hand, the efficiencies of the PE are suboptimal and inconsistent on many occasions, despite numerous enhancement efforts [6–9]. It is possible that the existing DNA repair mechanisms cooperate less optimally to limit PE activities. Interestingly, different from the conventional, nickase-based PE, several versions of a nuclease PE (PEn, based on the nuclease-active Cas9) have also been developed [10–12]. More recently, we and others have shown that employment of a PEn together with chemical or protein regulators of DSB repair could markedly enhance the rate of accurate edits [13, 14]. In our system named ubiquitin variant-assisted PEn (uPEn), an engineered ubiquitin variant known as i53 [15] acted via inducing DSB 5′-end resection to facilitate precise alignment of RT-synthesized single-stranded DNA (ssDNA) with the homologous downstream sequence [with reference to the protospacer adjacent motif (PAM) strand] of the DSB [14]. Benchmarking experiments showed that uPEn installed accurate edits at significantly higher rates than several advanced PE (nickase) forms and the Cas9/HDR platform, despite its remaining liability of causing certain levels of targeted indels [14]. Collectively, these recent developments have gradually unveiled the promising application potentials for PEns.

The incoming nick position by the PE (or the cleavage position by PEn toward a blunt end DSB) is located 3 nt upstream of the NGG PAM. As the RT module acts to catalyze a primer extension-type reaction at the exposed 3′-OH of the NTS, PE’s editing window is located downstream of the nick position (with the first position routinely specified as “+1″). Importantly, it is widely recognized that the editing rate of the nickase PE is negatively correlated with the nick-to-edit distances, so that to install a “distal edit” with its first new base at beyond the +6 location is generally challenging [16, 17]. Given the non-saturating nature of PAM availability (average of one NGG per 8 bp length considering both strands), it may be estimated that a significant percentage of hypothetical PE target positions are situated outside of favorable editing windows. Although a PAM-flexible uPEn version might be adapted to precisely place the cleavages, this countermeasure would be hampered by the often inferior activities of designer Cas9 modules [18, 19]. Such a practical issue for the editing window significantly hinders the applicability of a PE, especially in contexts that require precise edit installation at fixed genomic positions (e.g. for correction of pathogenic mutations).

Whether the PEns are liable to similar restraints of edit-to-cleavage distances have not been formally investigated. Indeed, during the initial development/characterization of the uPEn [14], we focused on edits adjacent to the cleavage positions. In the present study, we provide evidence that for those “distal edits” that are refractory to the state-of-the-art nickase PE, i.e. PE7 [20], the standard uPEn also becomes ineffective. To subsequently expand the editable space for uPEn, we develop new strategies of opposite strand [target strand (TS), instead of NTS] editing to enable edits upstream of cleavage positions. Such new upstream-modifying uPEn forms (3.1/3.2) drive efficient prime edits at a panel of originally refractory locations. Additionally, we reveal unique insights on the cellular responses affecting uPEn's TS editing efficiencies. Taken together, our work establishes universal tactics to effectively broaden the editable space of PEns, which will be instrumental for various future applications.

## Materials and methods

### DNA construction

The complete sequences of the PEn and uPEn plasmids and of some key constructs are listed in the Supplementary Notes section. To construct various guide RNA plasmids, including pegRNA, sgRNA, ActRNA:t (accessory template RNA for modifying target strand (ActRNA:t), pegRNA with a PBS mutation, and tsp-pegRNA (target strand-programming pegRNA), different polymerase chain reaction (PCR) strategies were employed based on the size of the DNA fragments. For the PCR amplification of products > 1 kb, the Phanta Flash Master Mix (Vazyme) was utilized. For fragments < 1 kb, the Phanta Max Master Mix (Vazyme) was employed. The guide RNA plasmid backbone was amplified from pGL3-U6-sgRNA-EGFP (Addgene #107 721). The assembly of the various pegRNA or sgRNA cassettes was conducted by preparation of annealed primers, followed by a step of cassette assembly into the backbone vector aided by the MultiF Seamless Assembly Mix (ABclonal). The assembly reactions were conducted in a total volume of 20 μl, comprising 10 μl of the enzyme mix and the longer and shorter fragments [in respective amounts (ng) of 0.02 × number of bases and 0.04 × number of bases]. The general primers utilized for construction are listed in Supplementary Table S1. The various types of pegRNA and sgRNA sequences utilized in this study are listed in the Supplementary Data (supplementary data.xlsx).

### Cell culture and transfection

HEK293T, U2OS, and HeLa cells were cultured in Dulbecco's modified Eagle's medium (DMEM) (Gibco) supplemented with 10% (v/v) fetal bovine serum (FBS) (Gibco). The cells were incubated at 37°C with 5% CO_2_ and passaged every 2–3 days when they reached ∼ 80% confluency. HepG2 human hepatocellular carcinoma cells and HepG2 cell-specific medium were purchased from Wuhan Pricella Biotechnology Co., Ltd. Cells were seeded into T25 flasks and maintained with HepG2 cell-specific medium. For transfections, cells were seeded in 24-well plates. HEK293T cells were transfected using EZ Trans reagent (Life-iLab) ∼ 16–24 h after seeding, at ∼ 60% confluency. Similarly, HeLa, U2OS, and HepG2 cells were transfected at the same confluency using Lipofectamine 3000 reagent (Thermo Fisher Scientific). For sgRNA and ActRNA:t experiments, cells were transfected with 900 ng of uPEn plasmid, 300 ng of sgRNA plasmid, and 300 ng of ActRNA:t plasmid. For pegRNA and tsp-pegRNA experiments, cells were transfected with 900 ng of uPEn plasmid, PE7 plasmid, or PEn plasmid, and 300 ng of pegRNA plasmid or tsp-pegRNA plasmid. To test the effect of the La protein module [20] on uPEn editing, the HEK293T cells were co-transfected with the La (1–194)-encoding plasmid (500 ng) and the uPEn components. For Cas9/HDR editing with an ssDNA template, cells were transfected with 900 ng of cas9 plasmid, 300 ng of sgRNA plasmid, and 50 ng of ssDNA donor template. The sequences for ssDNA templates are listed in Supplementary Table S2. To test the effect of DNA-dependent protein kinase (DNA-PK) inhibition on tsp-pegRNA-adapted PEn/uPEn, cells were pre-treated with 1 μM AZD7648 (MCE) and were subsequently transfected. For measurement of cell viability after AZD7648 treatment, the CellTiter-Lumi Plus Luminescence Cell Viability Assay kit from Beyotime (Shanghai, China) was used.

### Flow cytometry

Three days after transfection, cells were harvested for flow cytometry sorting using a BD Aria III. The sorting process was based on the presence of enhanced green fluorescent protein (EGFP) labeling on the pegRNA, ActRNA:t, or tsp-pegRNA plasmids and, occasionally, based on blue fluorescent protein (BFP) labeling on the sgRNA plasmids. For groups indicated by a single EGFP marker, cells were sorted using the EGFP^+^ gate. For groups indicated by both EGFP and BFP markers, cells were sorted using the EGFP^+^BFP^+^ gate. A total of 10 000 positive cells were collected by fluorescence-activated cell sorting (FACS) for subsequent genomic DNA preparation.

### Sample preparation for NGS

The cells were harvested and subsequently transferred to a solution of 50 μl of cell lysis buffer. The composition of the solution was as follows: 50 μl of 1 M Tris–HCl (pH 8.0), 25 μl of 10% sodium dodecylsulfate (SDS), and 200 μl of 20 mg/ml protease K. The remainder was constituted by water to a total volume of 5 ml. The cells were then lysed at 37°C for 1 h, followed by incubation at 80°C for 30 min. Subsequently, the target loci [or predicted off-target (OT) sites] were amplified by PCR, and subjected to next-generation sequencing (NGS).

### NGS analysis of amplicons

For analyses of the same sites edited under different experimental conditions, the 5′-primers with distinct barcodes were used for the preparation of the amplicons. The primers used for amplification of the target sites are listed in the Supplementary Data (supplementary data.xlsx). The primers were designed so that the Cas9 cleavage sites would be located close to the center of the amplicons. The touchdown PCR protocol was used, with an annealing temperature range of 65–57°C and a gradient of 0.5°C for 16 cycles, followed by 20 cycles of conventional amplification. Reactions were performed in a 40 μl reaction volume using 2× Phanta Max Master Mix (Dye Plus) (Vazyme). PCR products with different barcodes were pooled together, and the amplified DNA products were purified with the DNA Recovery Kit (Vazyme) according to the manufacturer′s instructions. OT site prediction was performed using the Cas-OFFinder tool [21]. The targeted NGS results were analyzed with the following process [5]. The deep sequencing experiments were carried out using the Illumina NovaSeq platform (PE150) at Genewiz or Annoroad Gene Technology. Sequencing reads were demultiplexed using MiSeq Reporter (Illumina). Alignment of amplicon sequences to a reference sequence was performed using CRISPResso2 [22]. For all NGS analyses, CRISPResso2 was run with quality filtering whereby only reads with an average quality score of at least 30 were considered. To quantify point mutation editing, CRISPResso2 was run in standard mode with the ‘discard_indel_reads’ option enabled. The editing frequency was determined by dividing the number of non-discarded reads containing the edit by the total number of reads aligned with all amplicons. For insertion or deletion edits, CRISPResso2 was run in HDR mode with an additional parameter “e” that corresponded to the supplement of a sequence of the desired, edited amplicon. The ‘discard_indel_read’ option was also enabled. Editing efficiency was calculated as the percentage of HDR aligned reads divided by the total number of reads aligned with all amplicons. For all experiments, the indel frequency was calculated as the number of discarded reads divided by the total number of reads. The indel quantification windows were set to extend 30 bp. For sequence alignment, the plot_window_size was adjusted according to the combined sizes of the insertion and the HDR. The corresponding imperfect editing rate was calculated as a percentage of reads with imperfect HDR within the total number of reads aligned with all amplicons. For long-read sequencing analyses, PCR amplification was performed using 2× Phanta UniFi Master Mix (Dye Plus) (Vazyme) with dual-index barcodes incorporated into both forward and reverse primers. Touchdown PCR conditions were optimized for 3 kb amplicons, starting with an initial annealing temperature of 68°C with a 0.5°C decrease per cycle for 16 cycles, followed by 20 cycles at 60°C. Purified PCR products from distinct barcodes were pooled in equimolar amounts and further purified using the FastPure Gel DNA Extraction Mini Kit (Vazyme) according to the manufacturer's protocol. DNA samples were sequenced via the PacBio HiFi platform (Genewiz). Demultiplexing of raw HiFi reads was performed with lima (v2.7.0) using dual-index barcodes, followed by alignment to target region references via pbmm2 (v1.13.0). Sequencing depth was analyzed with samtools (v1.19.2), and coverage plots were generated using R (v4.2.3).

### Off-target analysis

Potential OT sites were predicted in the human genome (GRCh38/hg38) using Cas-OFFinder(21) (version 2.4) (http://www.rgenome.net/cas-offinder). The sequences flanking the predicted OT sites were amplified with Phanta Max Super-Fidelity DNA Polymerase (Vazyme) and subjected to high-throughput sequencing using the Illumina NovaSeq platform (PE150) at Genewiz. The primers utilized for OT amplification are provided in the Supplementary Data (supplementary data.xlsx). Subsequently, the sequencing files were analyzed with CRISPResso2 [22] (version 2.2.11). The reads were aligned to the corresponding OT reference sequences using CRISPResso2 in standard batch mode with the following parameters: “-q 30″, “-w 20″, and “–discard_indel_reads”. Single nucleotide variations occurring at frequencies < 0.1% of the total reads within a sample were excluded from further analysis. The proportion of off-target edits was calculated as the percentage of reads containing indels divided by the total number of reads aligned across all amplicons.

### Bioinformatic survey of ClinVar-documented pathogenic SNPs for potential coverages

Single nucleotide polymorphisms (SNPs) corresponding to the GRCh38 reference genome were directly selected from the ClinVar variant dataset [23]. Duplicate variants were removed based on RS# (dbSNP) followed by clinical significance-based filtering to retain pathogenic variants. Coverage analysis on the SNP dataset was conducted via a PPSID module (adapted from https://github.com/huang-0323/PE-stop-finder), in combination with custom R pipelines [24].

### Statistics and reproducibility

All quantitative sample measurements were conducted using three parallelly transfected biological replicates (or two replicates in a few incidences, as indicated specifically in the figure legends). In certain instances, the results of utilizing a particular editing platform across multiple sites were summarized to provide a comprehensive account of the findings. The data were analyzed using GraphPad Prism v.8 (v.8.0.1). Data are presented as means ± SD as indicated in the figure legends. Differences (*P*-values) between groups were determined by two-tailed Student's *t*-tests.

## Results

### The need and the design framework for expanding the editable space by uPEn

Based on the prototype PEn, we recently adopted a ubiquitin-like helper module [15] to promote correct installation of RT-dependent edits, and developed the high-activity uPEn tool [14]. Here, we aimed to further refine uPEn toward a universally applicable platform for installation of small-sized edits. As the canonical PE presents low activities for distal edits (first intended new base at greater than the +6 position) [16, 17], it would be important to also determine the performances of uPEn for such potentially challenging tasks (Supplementary Fig. S1A). To this end, we designed a series of pegRNAs against various genomic loci, each specific for small insertional edits either at relatively distal positions (+7 to +9 positions), or at proximal positions (similar to those targeted previously [14]) (Supplementary Fig. S1B, C). A routine length of ∼13 bp was adopted for the PBS region of pegRNA. Moreover, a ∼20 bp homologous sequence was placed at the 5′ side of the edit-encoding sequence in the RTT [14], to aid accurate installation of edits. HEK293T cells were transfected with the plasmids encoding uPEn and respective pegRNAs. For the distal edits (Supplementary Fig. S1B), the same pegRNAs were transfected together with a very recent nickase PE (PE7) as a control [20]. The genomic DNA samples were subjected to targeted NGS analyses. Indeed, we found that even the state-of-the-art PE7 tool was ineffective in installing the distal edits (Supplementary Fig. S1B, left). Notably, a similar inactivity was also observed with uPEn for the same distal edits, as the editing outcomes were dominated by the undesirable products (Supplementary Fig. S1B, right). In contrast, for edits into more proximal positions (+1 to +4), uPEn was confirmed to exhibit robust prime-editing activities, along with its concomitant induction of certain on-target errors (Supplementary Fig. 1C) [14]. The editing rates by uPEn at the latter favorable positions also consistently exceeded those by PE7, as our test sampled edits (5, 7, or 8 bp insertions) that could be challenging for PEn [17, 25]. Therefore, despite the potent efficiencies of uPEn on proximal edits, our results underscored uPEn's lack of activity against distal edits, which also represents a common challenge for all existing PE platforms.

One potential explanation for the inefficiency of uPEn for distal edits is the possibility of recursive cleavage after a successful round of precise editing, due to the unaltered spacer and the PAM motif in the post-edit sequence (see schematic demonstrations in Supplementary Fig. S1A). Therefore, we closely analyzed the allele types associated with uPEn application for distal edits (Supplementary Fig. S1D–F). Notably, the great majority of undesirable edits were in the indel category and did not contain reverse-transcribed sequences. In addition, within the small fraction of RT-dependent edits (presently referred to as “prime edits”), the downstream, sequence duplication-type errors characteristic of older PEn versions were less common [10–12], consistent with the adoption of the i53 module in uPEn [14]. Although certain reads containing the correct insertions together with the upstream indels (indicative of secondary editing) could be detected, the overall low levels of prime edits in these samples assigned recursive cleavage as only a minor hurdle for uPEn activity toward distal edits. In retrospect, our results from the above uPEn applications displayed some resemblance to those from previous CRISPR/HDR experiments [26]. We speculate that the cellular DNA repair may tend to reject a DSB-originated homologous single-strand sequence that bears mutation(s) relatively distant from the initial DSB, leading to indel formation and/or lower editing rates (see schematic demonstrations in Supplementary Fig. S1A).

**Figure 1.**
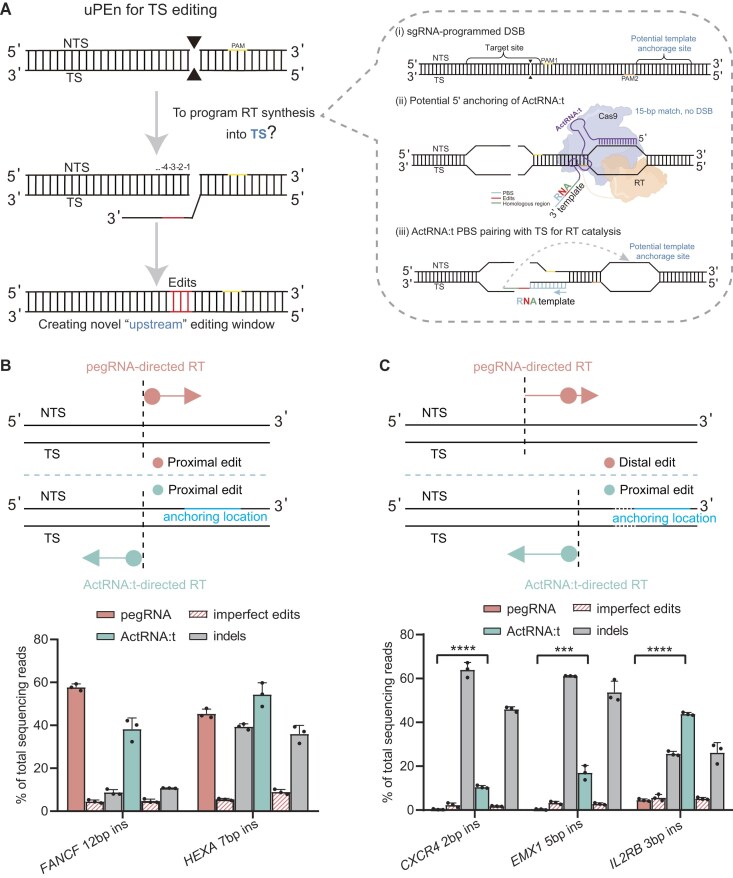
Development of an accessory template RNA for modifying target strand (ActRNA:t) to apply together with a cleaving sgRNA in a TS-editing derivative form of uPEn. (**A**) The scheme for adapting uPEn to TS editing. The processing of the DSB toward potential primer extension at the TS and the eventual installation of an edit are depicted on the left. Both the NTS and TS are marked. The positions of potential edits on the upstream end of DSB begin from “–1″. Unless indicated otherwise, the uPEn expression construct (PEn + i53) was used for the experiments. We herein describe the corresponding TS-editing protein complex(s) under the term “uPEn”, although the regulatory action of i53 is not routinely shown in the schemes. As uPEn would be more effective for more proximal edits, TS modification by uPEn would complement the conventional NTS editing framework to substantially expand the editable space. In a more detailed view, the initial configuration of the ActRNA:t accessory component for programming TS editing is depicted in the right “zoomed-in” region. A cleaving sgRNA (instead of pegRNA) directs uPEn to generate a DSB. A pegRNA-like architecture was adopted for constructing the ActRNA:t. It contains a shortened 15 bp spacer (without causing cleavage) against an opposite strand sequence near the sgRNA target site, for its potential anchorage to aid the ensuing actions. The 3′-extension of the ActRNA:t sequence features alternatively configured PBS + RTT according to the TS (but not NTS) at the upstream uPEn/sgRNA-dependent DSB. The coordinated actions by sgRNA and by ActRNA:t direct uPEn towards synthesizing edits into the TS. (**B**) The insertional edits directly next to the cleavage sites were first considered, so that the corresponding NTS and TS modification would be at proximal positions (marked by faint red/blue filled circles in the upper scheme). The pegRNA (NTS editing) and sgRNA (TS editing) had the same spacer (dotted lines showing cut positions). For TS editing, ActRNA:ts were designed to potentially bind to sequences (“anchoring location”) with the corresponding PAMs ∼ 10 bp downstream of cleavage positions. HEK293T cells were transfected with uPEn/pegRNA or uPEn/sgRNA/ActRNA:t (lower part). The targeted sites were subjected to NGS analyses (*n* = 3 biological replicates, mean ± SD). The accurate edits, as well as the imprecise prime edits and the indels from both groups, are presented. (**C**) Several target locations (*n* = 3 loci) were selected for being situated proximal to their downstream but not their upstream cleavable positions (relative positions illustrated by dotted lines in the upper scheme). Therefore, NTS and TS modifications, respectively, correspond to editing at distal (filled circle in faint red) and proximal positions (in faint blue). ActRNA:ts were designed to potentially bind to sequences (“anchoring location”) with the corresponding PAMs ∼ 30–60 bp downstream of cleavage positions. The experiments were conducted and analyzed similarly to as in (B). The results are presented in the lower bar graphs (*n* = 3 biological replicates, mean ± SD). The differences in the levels of accurate edits between the sgRNA/ActRNA:t and pegRNA groups were determined using two-tailed Student's *t*-test (***P < 0.001, ****P < 0.0001).

Such an issue regarding uPEn's editing window is also intrinsically attributed to its unidirectional “writing” activity (similar to that for a PE), due to the fact that the RT is programmed to only drive synthesis into the cleaved NTS. In the face of such an intrinsic limitation, we conceptualized that to also empower uPEn with TS-modifying activity could possibly lead to “unfolding” of its editing window (Fig. 1A, left). This may potentially convert some previously refractory editing locations into favorable locations dependening on their proximities to the downstream cleavable positions. Intriguingly, such a design principle has not been formally exemplified in the existing literature.

### Development of an accessory template RNA for modifying target strand (ActRNA:t) to apply together with a cleaving sgRNA in a TS-editing derivative form of uPEn

As the uPEn construct (for co-expression of PEn and the diffusible i53 helper module [14, 15]) would be used throughout the study, for simplicity we herein describe the corresponding TS-editing protein complex(s) under the term “uPEn”. In contrast to the canonical pegRNA to specify RT-dependent synthesis into the NTS, prospective TS modification by uPEn would require an alternatively configured designer template RNA. Moreover, as uPEn-dependent DNA cleavage requires guide RNA–TS pairing, the initial targeting process might potentially limit the TS accessibility for the prime-editing stage. Therefore, to resolve the problem, we considered separating the guide and template functions of the designer RNA into two molecules, i.e. a cleaving sgRNA and an accessory template RNA.

We next devised a strategy centered on the design of an ActRNA:t. It was considered that ActRNA:t would present two major features. First, it could potentially be anchored in the proximity of a uPEn/sgRNA-induced DSB. Secondly, it should contain a specialized template region to “collaterally” direct RT to copy into the TS of the DSB. Herein, a pegRNA-like architecture was adopted for constructing the ActRNA:t, with a shortened 15 bp spacer sequence to potentially anchor (without causing cleavage [27, 28]) downstream and on the opposite strand of the sgRNA target site [Fig. 1A, right-(ii)]. Furthermore, the 3′-extension sequence of ActRNA:t would feature alternatively configured PBS + RTT, corresponding to the sequence of TS at an upstream uPEn/sgRNA-dependent DSB (Fig. 1A, right).

We envisaged that the same DSB induced by the standard uPEn/pegRNA or, alternatively, by a currently designed uPEn/sgRNA (for applications involving ActRNA:t) could be subsequently exploited by the RT component to drive identical cut-site insertions (at position +1 and –1, respectively). Such cut-site insertions in either direction correspond to “proximal edits”, which would feature more robust rates suitable for detection (Fig. 1B, upper). Therefore, we first constructed the sgRNA/ActRNA:ts and pegRNAs for identical cut-site insertions (at two genomic loci) (Fig. 1B, lower). The anchoring locations (relevant PAMs) of the ActRNA:ts were specified to be ∼ 10 bp downstream of the sgRNA-associated DSBs. The 3′-extension sequences for the ActRNA:t and standard pegRNA were designed with the same rule (∼ 13 bp PBS and ∼ 20 bp homologous sequence in RTT), but in accordance with the TS and NTS sequences, respectively. Next, HEK293T cells were transfected with the plasmids for uPEn/sgRNA/ActRNA:t, or with those for standard uPEn/pegRNA. Quite encouragingly, this initial test yielded positive results. The sgRNA/ActRNA:t combo was clearly sufficient to direct uPEn toward TS modifications, which led to efficient installation of desirable cut-site insertions (Fig. 1B, lower). Besides the correct prime edits, uPEn/sgRNA/ActRNA:t also caused low percentages of imprecise prime edits and variable levels of indels. The general product patterns between the sgRNA/ActRNA:t and pegRNA groups were similar for such applications of cut-site editing (Fig. 1B, lower). These results clearly demonstrated the feasibility of adopting a specialized designer RNA component to enable uPEn to also modify the TS. In accordance with our previous nomenclature for the standard uPEn platform applied here (uPEn3) [14], we designated this sgRNA/ActRNA:t-guided version as uPEn3.1.

We next investigated whether sgRNA/ActRNA:t-dependent enablement of direct TS modification could lead to “unfolding” of uPEn's editing window and the conversion of some previously refractory editing locations into more favorable locations (Fig. 1C, upper). To this end, we selected several edits (three genomic loci) located proximal to their downstream but not upstream cleavable positions, for customized small insertions (Fig. 1C, lower). The sgRNA/ActRNA:t and pegRNA specified, respectively, by the downstream and upstream cleavage sites were constructed for each target location. For the sgRNA/ActRNA:t combos, the potential ActRNA:t anchorage sites downstream of the sgRNA-directed cleavage position were semi-randomly chosen (ActRNA:t-related PAMs at < 65 bp from the cleavages). In addition to the uPEn groups, as another control, the PE7 (with the pegRNAs) was also applied to attempt installation of the distal edits (Supplementary Fig. S2A). For all three target loci (*CXCR4*, *EMX1*, and*IL2RB*), while the standard uPEn/pegRNAs were largely ineffective for installing desirable edits (at 0.6, 0.5, and 4.5%, respectively), the sgRNA/ActRNA:t-adapted uPEn (uPEn3.1) showed markedly greater rates of accurate editing (at 10.5, 17.0, and 43.8%, respectively) (Fig. 1C, lower). Moreover, the latter rates by the TS-editing uPEn3.1 were also considerably higher than those by PE7/pegRNA (at 5.8, 3.1, and 19.5%, respectively), while PE7 expectedly featured substantially lower indels (see Supplementary Fig. S2A for comparisons). For both uPEn groups, the imprecise prime edits remained low in all samples. On the other hand, both uPEn approaches caused variable levels of indels at different loci (Fig. 1C, lower). The explicit illustrations of different allele frequencies between the two uPEn groups are presented (see Supplementary Fig. S2B and C for a representative site). In sum, these results have provided proof of principle that the combined options of applying uPEn with canonical pegRNA (uPEn3) as well as with sgRNA/ActRNA:t (uPEn3.1) could empower the tool with a substantially expanded editable space.

### Further characterization of uPEn/sgRNA/ActRNA:t-dependent TS editing (uPEn3.1)

**Figure 2.**
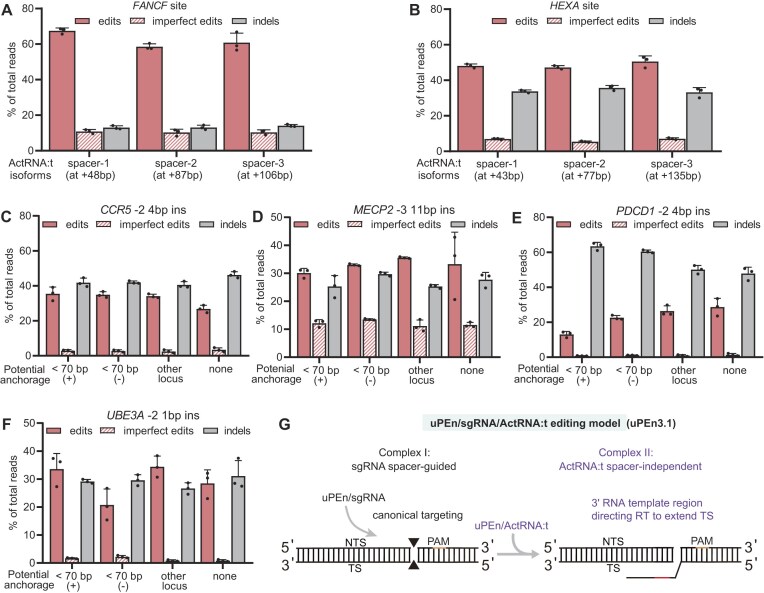
Further characterization of uPEn/sgRNA/ActRNA:t-dependent TS editing (uPEn3.1). (**A**, **B**) The sgRNA/ActRNA:t-adapted uPEn was applied to program insertional edits directly next to the cleavage sites. The ActRNA:ts were designed to potentially bind to various downstream sequences via the 15 bp spacers, with the distances between the corresponding PAMs and the cut position indicated on the graphs. HEK293T cells were transfected with uPEn/sgRNA/ActRNA:t. The targeted sites were subjected to NGS analyses. The accurate edits, as well as the imprecise prime edits and the indels, are presented (*n* = 3 biological replicates, mean ± SD). (**C**–**F**) The 15 bp spacers in several ActRNA:ts programming proximal edits were further diversified. Besides an ActRNA:t isoform designed to potentially bind downstream of the cut-site [< 70 bp (+)], such a “diversity series” also contained isoforms featuring 5′ spacers also corresponding to the DSB-neighboring upstream sequence on the TS [< 70 bp (–)], a sequence at an independent genomic locus (other locus), or an orthogonal sequence from sheep genome (none). The experiments and analyses were carried out similarly to as in (A) and (B) (*n* = 3 biological replicates, mean ± SD). (**G**) The deduced model for editing by sgRNA/ActRNA:t-adapted uPEn. The two-complex model is highly consistent with the experimental data. As the uPEn expression construct (PEn + i53) was used for the experiments, we herein describe the corresponding TS-editing protein complex(s) under the term “uPEn”. The regulatory action of i53 is not easily shown in the scheme.

Given the well-defined mechanistic model for nickase PE-driven primer extension at the NTS [5, 29], it was not clear how sgRNA/ActRNA:t-adapted uPEn could be organized structurally to instead drive direct modifications of the TS. In particular, how a collaterally anchored uPEn/ActRNA:t would engage synthesis of prime edits at a nearby DSB remained puzzling. To further probe the mechanisms underlying uPEn3.1-dependent TS editing, we considered examining whether the locations of the potential anchorage positions of ActRNA:ts would impact their activities. A series of ActRNA:t isoforms for insertional editing at two tested sites were further constructed (see Fig. 1B for reference). Each ActRNA:t isoform would potentially anchor (via a distinct 15 bp spacer motif) to a different location downstream of the intended cleavage (Fig. 2A, B). HEK293T cells were next edited with different uPEn/sgRNA/ActRNA:t combinations. The results showed that despite the differences in potential anchorage positions, the series of ActRNA:t isoforms with the same 3′ RT-instructing extension sequence largely presented equivalent activities for both accurate and imprecise edits (Fig. 2A, B; and see also Fig. 1B).

Although these results could have implied that the potential anchorage position for ActRNA:t was flexible enough near the DSB to sufficiently support subsequent reverse transcription, the lack of quantitative differences among parallel ActRNA:t isoforms appeared surprising. We therefore further diversified the 15 bp spacers in other ActRNA:ts (for proximal insertional edits at four genomic loci). Besides the ActRNA:t isoform designed similarly to that above (see Figs 1B and 2A, B), such a “diversity series” contained isoforms featuring 5′ spacers also corresponding to the upstream sequence near the DSB, sequence at an independent genomic locus, or an orthogonal sequence from other species (Fig. 2C–F). These ActRNA:t isoforms were applied together with uPEn/sgRNA to edit HEK293T cells. Notably, regardless of the choice of sequence for the 15 bp 5′ spacers, different ActRNA:t isoforms presented highly consistent activities for both accurate and unintended edits (Fig. 2C–F). These results indeed disprove our earlier inclination towards the role of potential 5′ anchorage near the DSB for ActRNA:t function (see Fig. 1A, right).

Together, the above data suggest a model whereby sgRNA/ActRNA:t-adapted uPEn (uPEn3.1) installs desirable prime edits via the sequential actions of two discrete complexes sharing uPEn protein (Fig. 2G, and see the legend for rationale on terminology). A uPEn/sgRNA complex (complex I) initially induces a targeted DSB via the Cas9 moiety. Next, the dissociation of complex I is most probably required to permit TS accessibility for the next stage. Afterwards, a second complex of uPEn/ActRNA:t (complex II) accesses the TS of the earlier DSB in an exclusively 3′ tail-oriented manner (independent of the 5′-spacer region in ActRNA:t) and catalyzes editing via the fused RT module (Fig. 2G). Although this two-complex model for uPEn-driven TS modification differs significantly from a standard single-complex editing mechanism for uPEn/pegRNA, it has suggested a straightforward mechanistic framework to interpret such a non-canonical activity.

### Configuration of a streamlined target strand-programming pegRNA (tsp-pegRNA) for a uPEn3.2 editor

**Figure 3.**
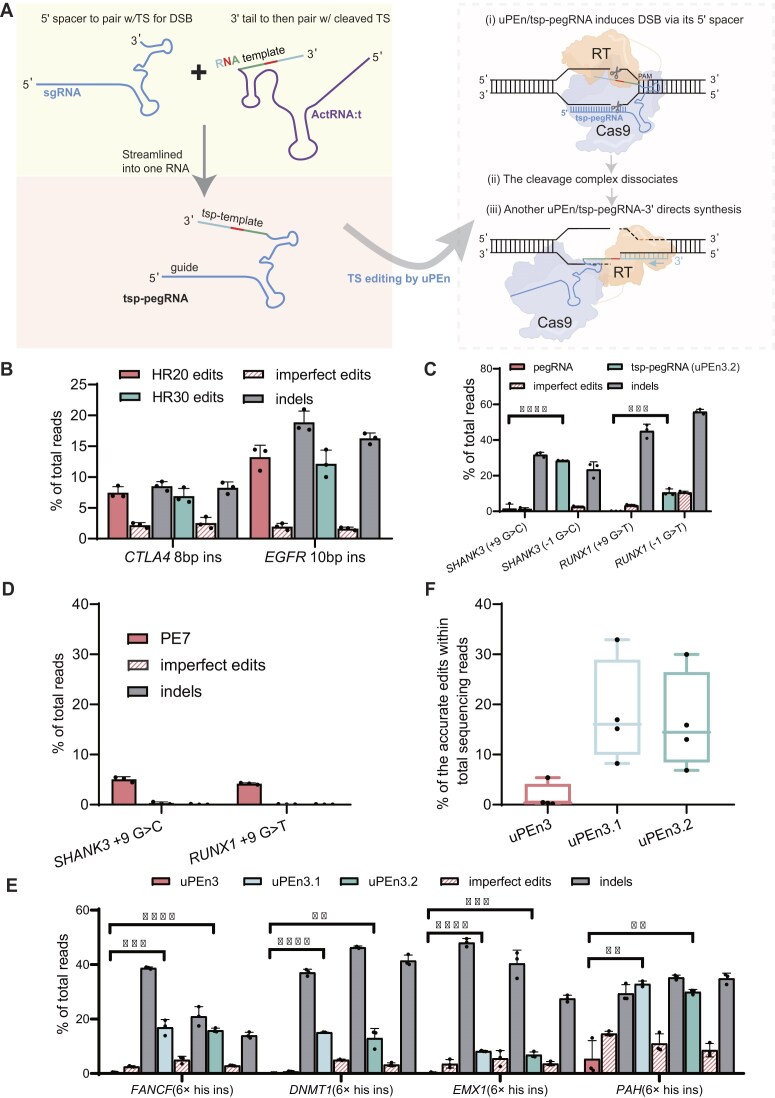
Configuration of a streamlined target strand-programming pegRNA (tsp-pegRNA) for an uPEn3.2 editor. (**A**) The scheme for devising the single-entity tsp-pegRNA is presented on the left. Given the two-complex model shown earlier for sgRNA/ActRNA:t-adapted uPEn, it appeared possible to streamline the dual-RNA combination into a single, yet non-canonical pegRNA (tsp-pegRNA). The PBS and RTT of the tsp-pegRNA bear complementarity to the TS sequence around its cleavage position defined by the 5′-spacer module. Analogous to the two-complex model for uPEn/sgRNA/ActRNA:t, two differentially oriented uPEn/tsp-pegRNA complexes may operate successively to drive TS editing (in the more detailed model shown on the right). (**B**) The tsp-pegRNAs were designed for programming cut-site insertions. For each edit, two tsp-pegRNA isoforms with a 20 and 30 bp homologous sequence were assembled within each RTT (PBS at ∼13 bp). The editing performances of each uPEn/tsp-pegRNA in HEK293T cells were determined (*n* = 3 biological replications, mean ± SD). (**C**, **D**) Different target edits were selected for being situated proximal to their downstream but not upstream cleavable positions. Therefore, NTS and TS modifications, respectively, correspond to editing at distal and proximal positions. In (**C**), the efficiencies of pegRNA- and tsp-pegRNA-adapted uPEn for two loci were compared (HEK293T cells, *n* = 3 biological replications, mean ± SD). The differences in the levels of accurate edits between the pegRNA and the tsp-pegRNA (uPEn3.2) group were determined using two-tailed Student's *t*-test (***P  < 0.001, ****P  <0.0001). In (**D**), PE7/pegRNA-dependent editing at the same two distal positions was carried out as a control (HEK293T cells, *n* = 3 biological replications, mean ± SD). (**E**, **F**) In (E), the efficiencies by pegRNA-, sgRNA/ActRNA:t-, and tsp-pegRNA-adapted uPEn (uPEn3, uPEn3.1, and uPEn3.2, respectively) for four additional loci were compared [HEK293T cells, *n* = 3 biological replications (except the *DNMT1*-ActRNA:t group with two replicates) mean ± SD]. Statistical analyses were performed similarly to (**C**) (**P < 0.01, ***P < 0.001, ****P < 0.0001). Results in (E) are further summarized in a box plot (F). The center line shows medians of all data points (*n* = 4 genomic loci), and the box limits correspond to the upper and the lower quartiles, while the whiskers extend to the largest and smallest values.

The two-complex mechanism underlying uPEn/sgRNA/ActRNA:t has shed light on a common framework to reconcile the two-way need for TS access by both the guide and template RNA modules to enable its subsequent modification. Therefore, we considered that it might be possible to streamline the TS-editing uPEn tool by reducing the two RNA components, i.e. sgRNA and ActRNA:t, back into a single, yet non-canonical pegRNA (Fig. 3A, left). Herein, the PBS and RTT of the pegRNA were designed to bear complementarity to the TS sequence, around its cleavage position defined by the 5′-spacer module. In essence, the 3′-extension sequence of such a non-canonical pegRNA features the opposite strand polarity in comparison with a canonical pegRNA. We named these alternatively configured pegRNAs as tsp-pegRNAs. We also noted that a recent preprint manuscript had introduced a similar design of TS-editing pegRNA architecture (termed “REDRAW”) to guide the Cas12a-derived PEn or with a Cas9 counterpart, achieving low correct editing activities [30]. Given our independent development of the sgRNA/ActRNA:t strategy and revelation of the underlying two-complex mechanistic model, we postulated that tsp-pegRNA-guided uPEn could act analogously to drive effective TS editing. Mechanistically, one uPEn/tsp-pegRNA complex would first induce a DSB at the target site, via the 5′-spacer-guided Cas9 activity. Following dissociation of this cleavage complex, a different 3′ tail-oriented uPEn/tsp-pegRNA complex would access the cleaved TS and would direct its RT module toward synthesis of edits (Fig. 3A, right). Though a potential contribution from the i53 module of uPEn to TS editing was speculated, a formal exploration will be described later in the study.

To test the above design principle of tsp-pegRNA, we selected two genomic loci and constructed tsp-pegRNAs for cut-site insertions (at –1 positions). The PBS sequences were set to be ∼13 bp. On the other hand, either a 20 bp or a 30 bp homologous sequence was assembled within each RTT (Fig. 3B). The editing performances of each uPEn/tsp-pegRNA combination in HEK293T cells were determined. Indeed, these tsp-pegRNAs enabled moderate rates of accurate insertions at the two genomic loci. Furthermore, the target sites also featured low levels of imprecise prime edits and variable levels of indels (Fig. 3B). These results provided evidence that uPEn coupled with a single-entity tsp-pegRNA is sufficient to program TS editing. As the current data also showed minimal differences in editing patterns between tsp-pegRNAs with either a 20 or 30 bp homologous sequence in RTT, the 20 bp homology cassettes were adopted to construct tsp-pegRNAs throughout the rest of the experiments. In line with the terminology of uPEn3.1 (with sgRNA/ActRNA:t), we designated the tsp-pegRNA-guided version as uPEn3.2.

Given the direct proof of tsp-pegRNA-dependent editing, we sought to examine whether a free RT module [31] (directed by tsp-pegRNA) could sufficiently drive the step of TS editing. Therefore, we tested tsp-pegRNA-dependent editing with either a full-length uPEn or a Cas9/RT-separated split format (Supplementary Fig. S3A). Notably, the split uPEn form featured markedly reduced activities for inducing desirable edits (into –1 or –2 positions, at three genomic loci), while showing only inconsistent changes in indel rates (Supplementary Fig. S3B). These results indicate that the tethering of full-length uPEn to tsp-pegRNA via its Cas9 moiety plays an important role to readily couple the RT activity with the template in order to drive effective TS modification. This notion is consistent with a previous study that explored a deliberate RT-tethering strategy for enhancement of the split canonical PE [32].

We next tested whether the adoption of tsp-pegRNA (uPEn3.2), like the earlier uPEn3.1 strategy (see Fig. 1C), could also help to convert some previously refractory editing locations into more favorable locations. The editing positions at two genomic loci were selected on the basis of their relative separations from the upstream cleavable position and, conversely, of their proximity to the downstream cleavable position (Fig. 3C, corresponding to modifications at +9 and –1 positions, respectively). The pegRNAs and tsp-pegRNAs were accordingly designed. Another editing test with PE7/pegRNAs was used as a control. As expected, while the standard uPEn/pegRNAs or PE7 were ineffective for introducing these distal edits (Fig. 3C, D), the alternative uPEn/tsp-pegRNA (uPEn3.2)-based proximal modification showed much enhanced rates (in HEK293T cells). On the other hand, both standard (uPEn3) and TS-editing uPEn3.2 induced variable levels of indels at different sites (Fig. 3C).

To directly compare the TS-editing performances of uPEn adapted with either tsp-pegRNA (uPEn3.2) or sgRNA/ActRNA:t (uPEn3.1), we applied the two current tools to install the same edits (6× His tag insertion) at positions that were considered distal for the standard uPEn, but proximal for the TS-editing forms. As an additional control, PE7 was applied together with pegRNAs (Supplementary Fig. S4). Indeed, PE7 was almost inactive for installing such distal insertions, which might also reflect a general challenge for PEs toward longer edits [17, 25]. Furthermore, compared with the ineffectiveness of uPEn/pegRNA (uPEn3) for insertion at such distal positions, both uPEn3.1 and uPEn3.2 strategies showed apparent activities to install accurate edits (Fig. 3E, F). Moreover, no significant differences in editing patterns were observed between the applications of uPEn3.1 and uPEn3.2. Such similarities also corroborated that these TS-editing tools adopt comparable two-complex mechanisms (see Figs 2G and 3A). We next extended the comparisons between uPEn3.1 and uPEn3.2 to various deletional and substitutional edits (Supplementary Fig. S5A, B). For deletional and substitutional edits, uPEn3.1 and uPEn3.2 overall achieved ∼30% correct editing rates, and performed similarly. In the rest of the work, we mainly focused on uPEn3.2, due to its operational simplicity.

### More extensive examination of the performance of uPEn3.2 in different cell lines

**Figure 4.**
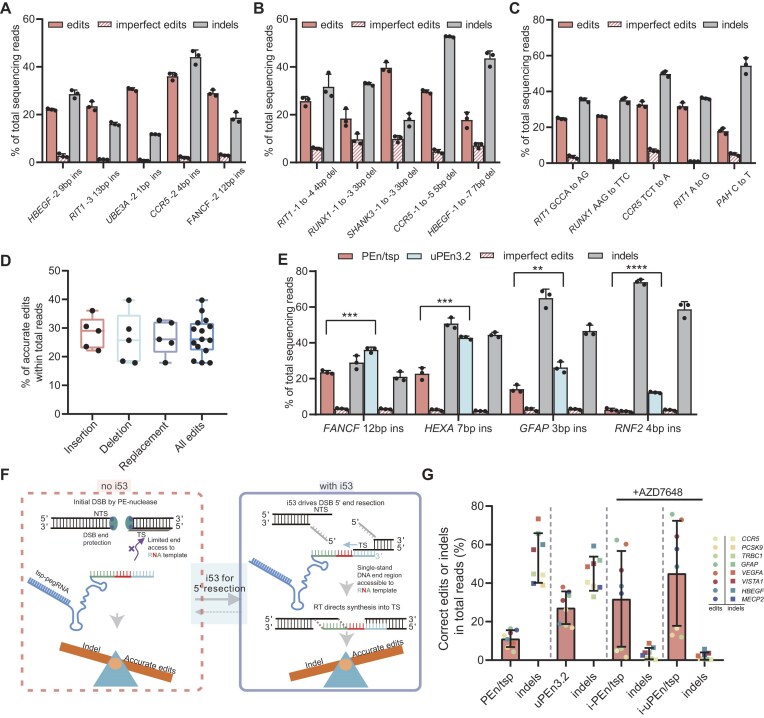
Further characterization of uPEn3.2 performances and associated cellular responses. (A–D) We systemically subjected uPEn3.2 (with tsp-pegRNAs) to installation of various small insertion-, deletion-, and replacement-type proximal edits [each for five sites, shown in (**A**), (**B**), and (**C**), respectively] in HEK293T cells (*n* = 3 biological replications, mean ± SD). The results in (A–C) are further summarized by the box plot in (**D**). The center line shows medians of all data points in the insertion- (five), deletion- (five), replacement- (five), and all-type groups (15). The box limits correspond to the upper the lower quartiles, while the whiskers extend to the largest and smallest values. (**E**) The effects of tsp-pegRNAs in combination with uPEn (with i53, uPEn3.2) and PEn (without i53, PEn/tsp) were compared for programming proximal edits at four genomic loci (HEK293T cells, *n* = 3 biological replications, mean ± SD). The differences in the levels of accurate edits between uPEn3.2 and PEn/tsp groups were determined using two-tailed Student's *t*-test (**P < 0.001, ***P < 0.001, ****P < 0.0001). (**F**) A model to explain the role of i53 in uPEn-dependent TS editing is presented. The relatively low accessibility of the TS 3′-OH end (in the duplex form) to PBS/tsp-pegRNA binding might present a major burden for accurate editing. The i53-promoted 5′-end resection of DSB and generation of a 3′-end ssDNA segment in the TS serves to enhance RNA binding (with PBS/tsp-pegRNA) and to license the initiation of reverse transcription. Therefore, i53 would preferentially drive uPEn/tsp-pegRNA-dependent prime edits in lieu of indels. (**G**) HEK293T cells were pre-treated or not with 1 μM AZD7648 for 3 h and were subsequently transfected with tsp-pegRNA-adapted PEn or uPEn (for eight different sites, *n* = 3 biological replicates). The targeted sites were subjected to NGS analyses. Data presented were derived from the same dataset as for Supplementary Fig. S16 (see Supplementary Fig. S16 for details of the edits). Measurements from the replicates were averaged. The correct edits and the indels at all sites are summarized in dot plot for a given condition. The tsp-pegRNA-guided PEn, uPEn, and their AZD7648 co-addition counterparts are marked as PEn/tsp, uPEn3.2, i-PEn/tsp, and i-uPEn/tsp, respectively. SD (±) for all data points in a column are also marked on the graph.

We next subjected uPEn3.2 to more extensive tests on installation of various small insertion-, deletion- and replacement-type proximal edits (each for five sites) in HEK293T cells (Fig. 4A–D). For all edit types in this series of experiments, uPEn3.2 resulted in consistent performances on accurate edits (17.8–39.7%, median 26%) and low levels of imperfect edits, while leading to more variable levels of indels (11.7–54.4%, median 35%). In the ensuing experiments, we applied uPEn3.2 for different types of edits in HeLa and U2OS cells (Supplementary Fig. S6A, B). In these two cell types (HeLa and U2OS), uPEn3.2 showed apparent precise editing activities, however at somewhat less pronounced levels [respective medians at 16% (12.5–22%) and 14% (10–21.5%)]. The indel rates were also more variable [respective medians at 15.8% (8.4–39%) and 26.5% (6.8–44%)] in HeLa and U2OS cells.

Given the present establishment of uPEn-dependent TS editing, we sought to compare other TS-modifying options with uPEn3.2 for editing performances. In this regard, the conventional Cas9/HDR method could also harness an ssDNA template complementary to the TS strand for programmed editing. Therefore, we prepared ssDNA editing templates with left/right 35 bp homology arms complementary to the TS sequence (for co-delivery with Cas9/sgRNA). Furthermore, for possibly editing at the upstream side (relative to the PAM) of a DSB, we considered an alternative uPEn strategy via the use of a variant but NTS-programming pegRNA bearing the small edits in the PBS portion. Subsequently, these tools were tested in HEK293T cells for editing performances at five different genomic loci (Supplementary Fig. 7A–G). The Cas9/HDR approach showed relatively low activities for precise editing, and mostly resulted in unintended edits. Interestingly, the variant, PBS-modified pegRNAs presented detectable, but low activities of precise editing, suggesting that certain incidences of 3′-end processing of NTS might possibly occur in the cells to allow infrequent incorporation of PBS-borne edits. In comparison with the use of these two low-activity tools, the application of uPEn3.2 was associated with markedly higher accurate edits (Supplementary Fig. S7A–E). These results clearly demonstrate the advantages of adopting uPEn3.2, over alternative options, for programming edits on the 5′ side of the cleavage site (in reference to PAM orientation) (Supplementary Fig. S7F, G).

We next searched for demonstrable metrics to exemplify the expanded PE editing scope given the present development of uPEn3.1/3.2. Based on general knowledge from previous applications [16, 17], we specified positions within 6 bp (+1 to + 6) downstream of the nCas9 (Cas9) cleavage point as a “favorable editing window” for PE and standard uPEn. Given that uPEn3.1/3.2 mediate upstream-directed editing, they could additionally cover the proximal positions between –1 and –6 relative to the cleavage point (“–6 bp window”). For demonstration purposes, we chose to focus on surveying the ClinVar-documented human pathogenic SNPs [23] for coverages by the +6 bp window and –6 bp window (Supplementary Data, in [supplementary data.xlsx]). We find that among all pathogenic SNPs, ∼39% are covered by the +6 bp window, and ∼34% are covered by the –6 bp window (Supplementary Fig. S8). There are also significant overlaps between the above two subsets of SNPs (∼20% of all surveyed SNPs, “covered by both”). Importantly, ∼14% of all surveyed SNPs are only covered by the –6 bp window, but not by the + 6 bp window, which implicates a unique opportunity for targeting such sites with the presently developed uPEn3.1/3.2. While the documented pathogenic SNPs only represent a small fraction of hypothetical uPEn targets, our data provide an unbiased outlook on the further expanded applicability of uPEn given the capability of TS editing.

For an immediate uPEn3.2 application, we focused on introducing pathogenic mutations in cultured cells, which would represent a key step for disease modeling. We chose two clinically relevant, pathogenic mutations in *TTR* (Thr80Ala) and *ASS1* (frameshift at the Phe150, “Phe150fs”) genes, causing hereditary amyloid transthyretin and type I citrullinemia, respectively [33, 34]. These genetic variations in *TTR* and *ASS1* presented challenges for standard uPEn (or PE) due to their relative distances from the upstream cleavable positions. However, being proximal to the downstream cleavable positions, they could be suitable for the TS-editing uPEn forms (Supplementary Fig. S9A). Consequently, we applied uPEn3.2 to installing *TTR*-Thr80Ala and *ASS1*-Phe150fs mutations in HEK293T cells. Indeed, transient transfection of uPEn3.2 components led to evident introduction of each variant (at > 15% rates), despite the occurrences of indel byproducts (at ∼ 20% rates) (Supplementary Fig. S9B). As the products of *TTR* and *ASS1* are expressed in hepatocytes, we also attempted transfection of the liver cell line HepG2 with uPEn3.2 to install these mutations (Supplementary Fig. S9C). Indeed, the patterns of correct and imprecise edits in HepG2 cells were similar to those in HEK293T cells. These results provide evidence that our TS-editing uPEn strategies have enriched the toolkit for practical genome-editing applications.

Given the current development of new uPEn forms that adopt either the sgRNA/ActRNA:t (uPEn3.1) or the tsp-pegRNA (uPEn3.2), we sought to evaluate their off-targeting characteristics. The potential OT sites corresponding to the sequences of the tested guide RNAs (against target sites within *FANCF*, *EMX1*, and *PAH*) were first nominated by Cas-OFFinder [21]. From the list of nominated sites, we then randomly selected several candidate OT sites featuring two, three, or four mismatches. One established OT site was also included in the *FANCF* group [35]. HEK293T cells were transfected with uPEn3.1 or uPEn3.2 against different target sites. The target and selected OT sites were subsequently analyzed by NGS (Supplementary Fig. S10). The on-target modifications, as well as the previously established OT hit (*FANCF* OT1) were confirmed. No off-targeting effects by uPEn3.1/3.2 were detected at another eight potential OT sites (Supplementary Fig. S10). Cross-referencing data from the same *FANCF* OT1 site in our previous study [14] revealed that both the standard and the current TS-programming uPEn platforms induced OT editing at similar magnitudes. These analyses implied that uPEn3.1/3.2 probably posed no greater off-targeting risk than standard uPEn.

In the context of PEns, it was previously demonstrated that a possible event of pegRNA-PBS binding to the off-target, homologous DSB end could result in unwanted priming and RT-dependent aberrant insertions [13]. The extent to which uPEn3.1/3.2 could also elicit such a type of OT editing needed to be addressed. However, as the tsp-pegRNA-PBS or ActRNA:t-PBS is complementary to the sequence downstream of the target cleavage site (–3 bp from the PAM), its sequence probably differs by greater degrees from the corresponding “primer” at the OT cleavage sites, potentially limiting RT-dependent insertions.

Therefore, we utilized NGS data from our previous work (uploaded at NCBI-SRA: RJNA847383) corresponding to PEn (uPEn) hits at the reference OT1 site of pegRNA-*FANCF* [14]. As the pegRNA-PBS presented two mismatches from the OT site “primer” sequence, we re-analyzed the data with CRISPResso [22], and concentrated on the rates of aberrant insertions. We found that edits by PEn/pegRNA at this OT site featured a significant portion of RT-dependent insertion products. In the parallel uPEn/pegRNA group, the percentage of such aberrant insertional OT edits appeared much lower, but was still discernible (Supplementary Fig. S11A, B). Next, we focused on the current NGS data at the same OT site (OT1-*FANCF* for both uPEn3.1/3.2, Supplementary Fig. S10). Importantly, while uPEn3.1/3.2 induced similar magnitudes (∼ 3.5%) of OT editing to standard PEn/uPEn, the current edits at this OT site by uPEn3.1/3.2 showed no RT-dependent insertions (Supplementary Fig. S11A, B). This was probably attributed to a total of five priming mismatches (for a 13 nt PBS in ActRNA:t or tsp-pegRNA) that prevented potential RT-driven insertion events at the OT site. Collectively, these analyses suggest that OT priming is likely to be less common for the TS-editing uPEn (uPEn3.1/3.2).

We further reasoned that quantitative assessment of how PBS base pairing stringency affects uPEn3.2 TS-editing outcomes would be important. To this end, we introduced single or double mismatches to the PBS region of tsp-pegRNAs and examined how such mismatches affected the performances of uPEn3.2 (Supplementary Fig. S12A). Overall, mismatches near the 5′ end of PBS were much less tolerable than those at the 3′ end (Supplementary Fig. S12B, C). For instance, the most 5′ PBS mismatch inhibited the prime editing rates of uPEn3.2 by ∼90%. Double mismatches located up to positions 5–6 from the 5′ border of PBS could nearly eliminate uPEn3.2-dependent prime edits, and those at position 9–10 could mitigate the levels of prime edits by ∼60%. These results demonstrate that for RT-dependent editing by uPEn3.2, a substantial homology between the PBS of tsp-pegRNA and the DSB end is required. Such specificity control will limit the risk of aberrant insertions not only at the OT sites, but also at any stochastically occurring DSB sites.

### Additional insights on the key cellular responses contributing to uPEn-dependent TS editing

The mechanistic model for TS-editing uPEn tools thus far appeared to suggest that a similar design involving the use of sgRNA/ActRNA:ts or tsp-pegRNAs could potentially be adopted to also engage TS editing by nickase PE. To probe such a possibility, we prepared a variant PE construct that carries a D10A mutation in the Cas9 module (PE-D10A) for initial nicking of the TS (Supplementary Fig. S13A). The targeting sgRNAs and the corresponding ActRNA:ts were constructed for installation of proximal edits (mostly into –2 positions) at various genomic loci (Supplementary Fig. S13B, C). The editing activities of sgRNA/ActRNA:t-adapted PE-D10A were determined in HEK293T cells. Interestingly, the results showed that the nickase TS-editing platform was inefficient at installing accurate edits (median of ∼ 5%), even though it appeared to retain the virtue of low error rates (Supplementary Fig. S13B, C). The compatibility of uPEn, but not the nickase PE, with TS editing implied that certain cellular responses associated with DSB repair might be important for uPEn3.1/3.2.

It is worth re-emphasizing that uPEn (but not the prototype PEn) employs the i53 helper module which is known to operate restrictedly upon DSB-induced repair processes [15]. Therefore, to unravel the cellular responses involved in uPEn-dependent TS editing, we chose to compare the performances of tsp-pegRNA-adapted uPEn and PEn. Four genomic sites were selected for installation of proximal edits by tsp-pegRNAs. The results from all sites demonstrated that uPEn3.2 induced edits which were more accurate, and inversely fewer indels than PEn/tsp-pegRNA (Fig. 4E). Consequently, uPEn3.2 enhanced edit/indel rates over PEn/tsp-pegRNA by > 2.3-fold (Supplementary Fig. 14A). On the other hand, both PEn and uPEn groups (with tsp-pegRNAs) contained minimal imprecise prime edits (Fig. 4E; Supplementary Fig. S14B). Therefore, for editing with both canonical pegRNA [14] and tsp-pegRNA (uPEn3.2, this work), the i53 module potently contributed to uPEn-dependent accurate edits. However, while i53 limited imprecise prime edits but not indels with canonical pegRNA [14], it instead specified an indel-reducing activity with tsp-pegRNA (Fig. 4E).

We reason that unlike the structurally reachable 3′-OH end in the NTS [29], the relatively low accessibility of the TS 3′-OH end (in the duplex form) to PBS/tsp-pegRNA binding might present a major hurdle for the subsequent TS modification (Fig. 4F, left). Such a challenge could be alleviated by i53 through induction of 5′-end resection at a DSB and generation of an ssDNA TS segment poised for RNA binding (with PBS/tsp-pegRNA) (Fig. 4F, right). The consequent RT-catalyzed TS extension at the DSB would readily oppose the error-prone NHEJ process [11, 14], which is highly consistent with the observed i53-dependent inverse changes in prime edits and the indels (see Fig. 4E). On the other hand, given the considerable strand accessibility on the upstream end of Cas9-induced DSBs [36, 37], it may be envisaged that the pairing of 3′-extended TS with the upstream homologous region can readily proceed even without i53 (corresponding to low imprecise prime edits, Fig. 4E). Overall, our data and model point to a unique and early rate-limiting step, i.e. 5′ resection-aided template RNA binding, for uPEn-dependent modification of TS (Fig. 4F).

Despite the beneficial action of i53, our TS-editing uPEn tools were still associated with considerable editing impurities mostly in the form of indels. It was previously shown that the 3′ portion of pegRNA is susceptible to degradation in cells, probably because this region is not bound and protected by the Cas9 module of PE, unlike the remaining part of pegRNA. This could lead to accumulation of unproductive, 3′-truncated pegRNA [38]. In the context of uPEn3.2, due to the generation of a DSB intermediate, accumulation of 3′-truncated tsp-pegRNA might not only inhibit correct edits, but also concomitantly increase indels. We therefore wondered whether improving the 3′ stability of the tsp-pegRNAs could potentially enhance uPEn-mediated TS editing. To this end, we tested a strategy similar to that in PE7 [20], and introduced the uPEn3.2 components together with the La (1–194) protein module for pegRNA protection (Supplementary Fig. S15). Interestingly, the La protein module did not promote correct edits or reduce the levels of indels. Although the current result did not show a significant impact of the La protein on uPEn3.2 performances, future investigation using alternative strategies is warranted to conclusively evaluate the potential of using 3′-stabilized tsp-pegRNA for enhancing uPEn-dependent TS editing.

As the NHEJ pathway was likely to contribute to uPEn3.2-induced indels, we utilized a well-characterized DNA-PK inhibitor (i.e. AZD7648) to inhibit the upstream events leading to NHEJ [39]. It was quite remarkable that co-administration of AZD7648 with tsp-pegRNA-adapted PEn or uPEn (“i-PEn/tsp” or “i-uPEn/tsp”) notably alleviated the unwanted indels (Fig. 4G; Supplementary Fig. S16A). Quantitatively, i-uPEn/tsp showed an average of a 43-fold improvement in edit:indel ratios over uPEn/tsp-pegRNA alone for various editing types (a similar 39-fold upgrade was achieved by i-PEn/tsp over the PEn counterpart) (Supplementary Fig. S16B). In contrast, the indel rates by standard NTS-editing uPEn were relatively refractory to AZD7648 co-administration (Supplementary Fig. S17). Although future investigations are warranted to explain such different consequences, these observations have highlighted a particularly robust effect of AZD7648 on improving the product purities of the currently developed TS-editing PEn and uPEn. With regards to the rates of correct edits, the i-uPEn/tsp group consistently outperformed i-PEn/tsp, further indicating a non-redundant role for i53 in conjunction with AZD7648’s action. Importantly, for some targeted edits, the editing rates by i-uPEn/tsp were the most potent among all tested nuclease PEs (± i53/± AZD7648) (Fig. 4G; Supplementary Fig. S16A), which strongly supported i-uPEn/tsp as a promising improvement strategy over uPEn3.2.

At the dose applied, AZD7648 showed slight toxicity, while the measured changes in cell viability did reach statistical significance (Supplementary Fig. S18). Besides the analyses of editing outcomes by small amplicon sequencing, it would be important to determine whether the presently adapted PEn (in various forms including uPEn3.2 and i-uPEn/tsp) is associated with the risk of inducing larger deletions [40, 41]. To this end, we carried out additional experiments to edit HEK293T cells at three different target sites by tsp-pegRNA-guided Cas9 and PEn, as well as by uPEn3.2 and i-uPEn/tsp. PacBio long-read sequencing was performed for the target loci. While small deletions could be observed in tsp-pegRNA-guided Cas9, PEn, and uPEn groups, the i-uPEn/tsp group presented very low levels of small deletions (Supplementary Fig. S19A–C), correlating to the high-purity editing pattern demonstrated for this tool (Fig. 4G; Supplementary Fig. S16A). Cas9/tsp-pegRNA also led to low but detectable levels of larger deletions in two out of three sites, while its PEn counterpart minimally induced such byproducts (Supplementary Fig. S19A–C). The latter observation was consistent with previous comparisons between Cas9 and PEn [13]. Like PEn/tsp-pegRNA, uPEn3.2 and i-uPEn/tsp caused overall negligible levels of larger deletions (Supplementary Fig. S19A–C). Collectively, the long-read sequencing results confirmed the favorable on-target genetic safety of tsp-pegRNA-guided PEn, even when it was applied together with DSB repair-regulatory agents including i53 (uPEn3.2) or i53 + AZD7648 (i-uPEn/tsp).

## Discussion

Cas9/HDR and the more recent PE systems constitute two major categories of genome-editing tools capable of inducing diverse types of programmed edits [3]. Subsequent to the establishment of nickase-based PE, several groups presented PEn as a hybrid system that merges the generation of DSB (Cas9 nuclease) with reverse-transcriptional synthesis of edits (PE/RT activity) to drive specific edits [10–13]. Based on the prototype PEn, we recently implemented a helper module (i53 [15]) for proper DSB repair, and consequently developed the high-potency uPEn system [14]. Indeed, uPEn was benchmarked to exhibit superior actual activities for correct edits to the advanced nickase PEs. Therefore, in spite of its association with on-target impurities, uPEn represents a promising editing platform for diverse applications [42].

One well-known limitation for the editable space of Cas9/HDR and PE is that their activities are inversely correlated to the cleavage-to-target distances [16, 17 ,26]. Herein, we observed that like the nickase PE, uPEn was also inefficient in installing distal edits (first new base > 6 bp away from the cleavage points) (Supplementary Fig. S1). The fact that the edits tested herein often featured sizes of from several to > 10 base pairs (as opposed to more undersized edits) might have further challenged the performances of standard uPEn (or nickase PE) at distal positions (see Fig. 3E and Supplementary Fig. S4 for examples of 18 bp insertions). Such an issue with uPEn's editing window would significantly hinder its potential for applications intended to modify fixed genomic positions (e.g. for precise alteration of defined genetic elements [43], or for correction of given pathogenic mutations [44]). We considered modifying another intrinsic restraint of uPEn, i.e. its NTS-only editing framework, and envisaged that to alternatively program RT-dependent synthesis into the TS might effectively “unfold” uPEn's editing window in the direction upstream of DSB (Fig. 1A).

We subsequently devised the ActRNA:t accessory RNA component to combine with the cleavage activity of a uPEn/sgRNA (Fig. 1A). The ActRNA:t has a broadly similar configuration to a pegRNA, but mainly serves the specialized role as the template for reverse transcription into the TS at a uPEn/sgRNA-programmed DSB (Figs 1 and 2). Our data further support an initially unanticipated two-complex model where uPEn distributes into independent complexes with sgRNA and with ActRNA:t, to sequentially drive cleavage and then TS editing (uPEn3.1). These promising developments inspired our further efforts to streamline the sgRNA component and the TS-modifying template RNA component into a bifunctional, target strand-programming pegRNA (tsp-pegRNA) (Fig. 3A). Importantly, like sgRNA/ActRNA:t, tsp-pegRNA also endowed uPEn with TS modification activities (Fig. 3). Herein, we envisage that the bifunctional tsp-pegRNA can engage uPEn into two differentially oriented complexes that successively access a TS to drive target cleavage and TS editing (Fig. 3, uPEn3.2). In consequence, both adaptations (uPEn3.1 and uPEn3.2) enabled successful modifications at a series of sites refractory to the standard uPEn as well as the nickase PE, and upgraded uPEn toward much expanded editable space (Figs 1 and 3; Supplementary Fig. S8).

While our work was in revision, we noted a very recent report that developed a dual NTS-nicking strategy (named “EXPERT”) to program PE edits on the 5′ side of the more downstream nick position [45]. This interesting design revolves around a re-engineered pegRNA (exp-pegRNA). The spacer of exp-pegRNA would specify the position of the downstream nick, whereas its 3′ PBS would bind to a parallelly induced, upstream nick on the same strand (NTS) to initiate RT-dependent synthesis. Consequently, such a collateral synthesis-dependent strategy enables prime edits upstream of the second nick, introducing a new editing window that may be alternatively reached by the present uPEn3.1/3.2 via opposite-strand (TS) synthesis at a single DSB. Given the differences in design principles, we envisage that EXPERT and the current uPEn3.1/3.2 represent complementary platforms to expand the application potential of PEs.

Systematic analyses of uPEn3.2 editing in several human cell lines demonstrated generally consistent activities for various edit types (Fig. 4A–D; Supplementary Figs S5 and S6). Importantly, we found that uPEn/tsp-pegRNA-dependent edit/indel ratios were positively impacted by its i53 moiety (Fig. 4E; Supplementary Fig. S14). These results reveal that the unseparated duplex DNA segment near the 3′-OH of the TS represents a major limiting step for tsp-pegRNA activity, whose alleviation is highly dependent on i53-induced 5′ resection (Fig. 4F). Such an insight is likely to be a key to future development of other TS-editing PEns. Indeed, the quite low levels of precise editing by the “REDRAW” TS-editing pegRNA strategy mentioned earlier [30] may indeed be due to the apparent lack of a co-delivered regulator (i.e. i53) to facilitate the initiation of reverse transcription. The same mechanism may also provide an explanation for the ineffective TS editing with the canonical PE (with a D10A nCas9) system (Supplementary Fig. S13), and suggests the potential path(s) toward future upgrades for the nickase PE.

To further reduce unwanted indels induced by uPEn3.2, we attempted to inhibit DNA-PK, a key player in the NHEJ pathway [46]. A DNA-PK inhibitor (AZD7648 [39]) was administered together with tsp-pegRNA-adapted PEn and uPEn to the cells (named i-PEn/tsp and i-uPEn/tsp, respectively). Notably, i-PEn/tsp and i-uPEn/tsp showed much improved editing purities in comparison with their no-AZD7648 counterparts (Fig. 4G; Supplementary Fig. S16). At some of the tested sites, i-uPEn/tsp and, to a less degree, i-PEn/tsp could drive substantially higher levels of correct edits in comparison with uPEn3.2, which supported future exploration of i-uPEn/tsp for diverse applications. Mechanistically, these results underscore a dominant role for the DNA-PK-dependent NHEJ pathway in causing indels upon the actions of tsp-pegRNA-guided PEn or uPEn. Intriguingly, in a previous report testing the effects of AZD7648 on standard PEn/pegRNA, the inhibitor did not effectively reduce the levels of direct indels, but enabled significant increases in desirable prime edits [13]. Our additional data also showed that the indel rates by standard NTS-editing uPEn were relatively refractory to AZD7648 co-administration (Supplementary Fig. S17). Therefore, we speculate that the cellular repair pathways underlying (u)PEn/tsp-pegRNA editing may present differences from those suggested for standard (u)PEn [13, 14 ,47]. Such a mechanistic insight warrants future investigations.

On the practical side, through “unfolding” uPEn's editable space (by enablement of its TS-editing capacity), our study has provided this potent editor platform with another key upgrade in regards to its versatility. In particular, we envisage that our latest uPEn toolkit (i.e. uPEn3, 3.1, 3.2, and i-uPEn/tsp) represents a multi-option resource toward installation of edits at fixed genomic locations, e.g. for therapeutic editing to correct given pathogenic mutations. Future efforts to test various uPEn forms in therapeutically relevant cells [44] and to improve uPEn's safety profile are needed. Along this line, adoption of high-fidelity Cas9 variants (see a recent review [4]) in uPEn represents a productive direction.

Overall, the present work demonstrates a series of developments to unfold uPEn's editable space via re-configuration of the template RNA architectures for TS editing. These strategies have enabled, for the first time, effective installation of upstream-directed prime edits. The continuously enriched uPEn toolkit will be instrumental not only to basic research, but also to biomedical and agricultural applications. Our study willll also stimulate future works to broadly advance PE technology.

## Supplementary Material

gkaf522_Supplemental_Files

## Data Availability

The targeted deep-sequencing data generated in this study have been submitted to the NCBI BioProject database (https://www.ncbi.nlm.nih.gov/bioproject/) under accession number PRJNA1160008.

## Supplementary data

Supplementary data are available at NAR online.
