# Supplementary material for: Configuration of adaptable template RNA architectures to unfold the editable space of a nuclease prime editor
Source: Nucleic Acids Res. 2025 Jun 11;53(11):gkaf522. doi: 10.1093/nar/gkaf522 (PMC12153339; doi:10.1093/nar/gkaf522)
Supplement: gkaf522_Supplemental_Files [file gkaf522_supplemental_files.zip › 050925 Supplementary Figures-Tables-Notes.pdf]

Chen et al., Configuration of adaptable template RNA architectures to unfold the editable space of nuclease prime editor

Supplementary Figures:

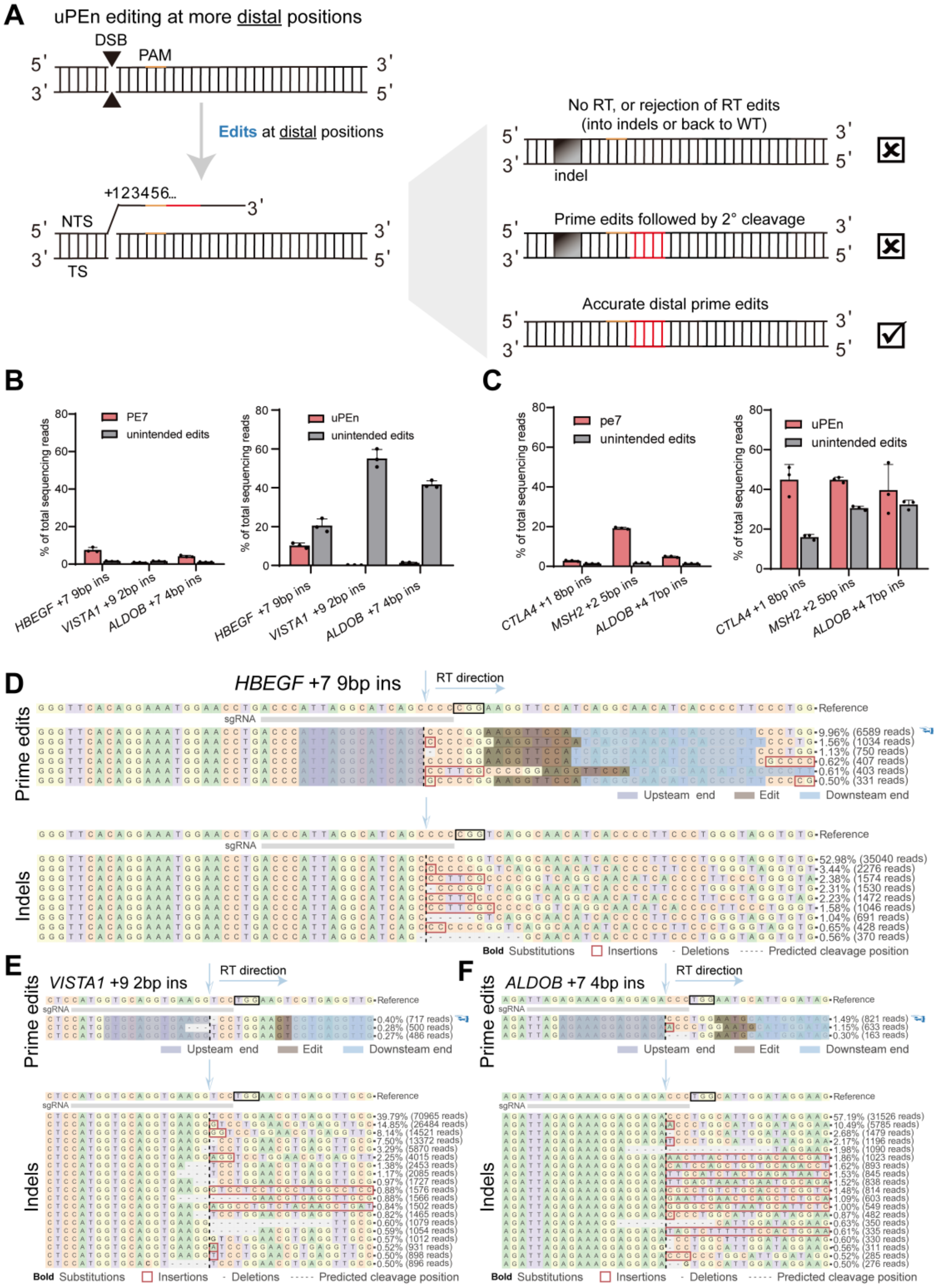

Supplementary Fig. S1: uPEn editing into positions more distal from the cleavage positions. (**A**) A schematic illustration of outcomes of uPEn/pegRNA-dependent editing into positions more distal from the cleavage position (marked in red). On the left, the scheme shows the DSB formation and RT-dependent extension of NTS. The potential editing products are illustrated on the right. Significant portions of the editing outcomes from such a task would be unwanted (marked by the boxes with a cross). Firstly, the nuclease-dependent DSB can either undergo NHEJ repair, or be subjected to catalysis by RT for templated extension in NTS. A direct NHEJ repair would lead to indel formation (☒). Moreover, given the nature of "distal" edits, the RT-extended ssDNA shall contain a 5′ homologous portion proceeding the intended edit (and the following 3′ homologous tail). Therefore, the cellular repair would tend to reject such a 3′-extended intermediate, and lead to either the NHEJ products, or to the repair of DSB into the WT allele. Secondly, there also exists the possibility that recursive cleavage can occur after a successful round of precise editing, due to the unaltered spacer and the PAM motif in the post-edit sequence. Such could lead to reads containing the correct edits together with upstream indels (☒). Consequently, for such challenging tasks, the rates of accurate prime edits (☑) may be compromised. (**B, C**) We designed pegRNAs for installing edits into either distal positions (B), or into proximal positions (C), as indicated under the graphs. HEK293T cells were transfected with uPEn/pegRNAs (or with PE7/pegRNAs). The targeted sites were subjected to NGS analyses (n = 3 biological replicates, mean±SD). The accurate edits, as well as unintended edits for all groups are presented. (**D-F**) The results from distal position editing at different genomic loci (see the right sub-panel of (B)) are further displayed in the allele frequency graphs. The downward arrow represents the cleavage position, and the PAM sequence is highlighted by a black box. The accurate alleles are marked by a hand sign. The major prime edits (accurate and imprecise) and the indel-type edits are displayed separately, as indicated. For the 'prime edits' category, all alleles with frequency ≥ 0.20% are displayed. For the 'indels' category, all alleles with frequency ≥ 0.5% are shown.

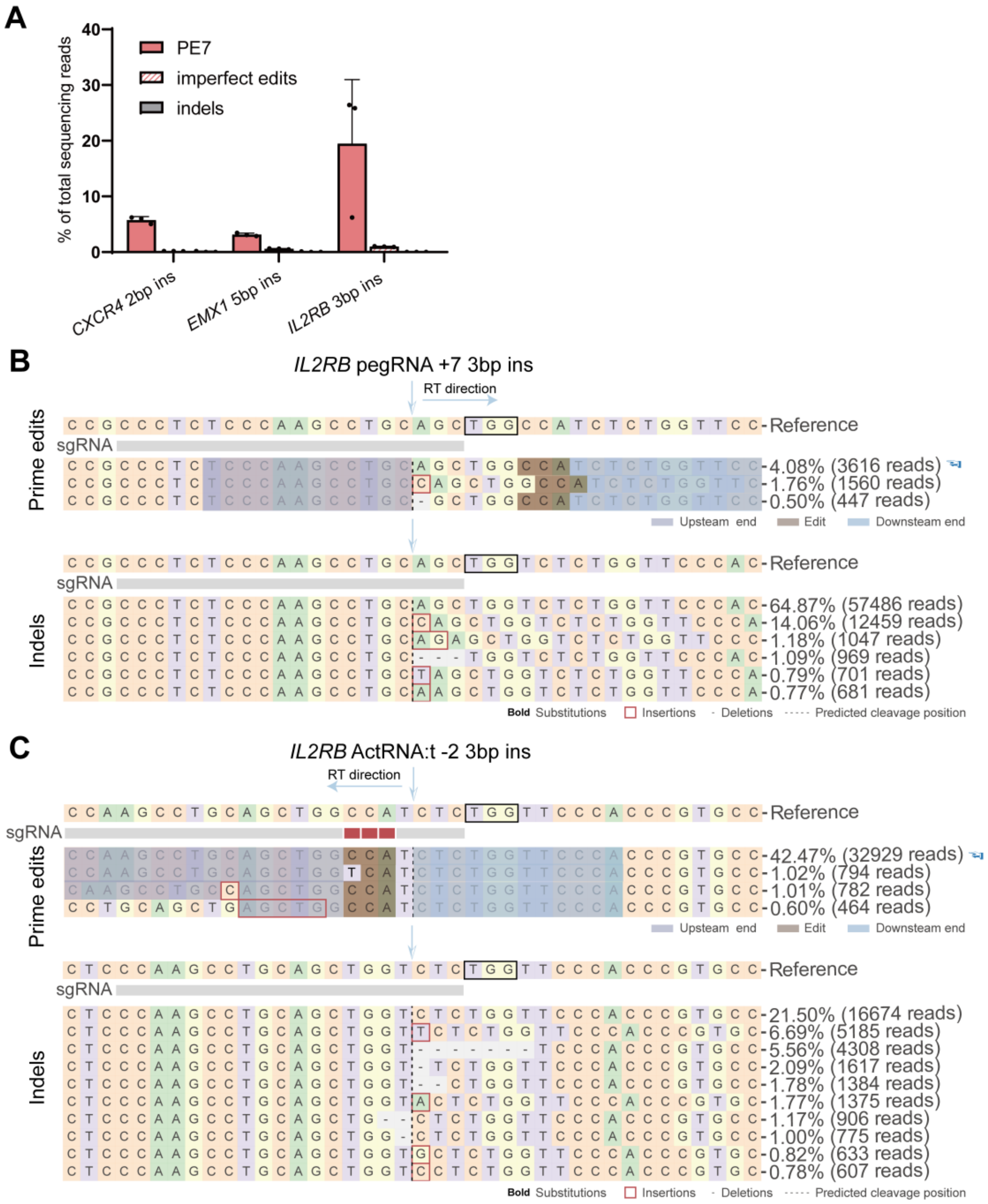

Supplementary Fig. S2: pegRNA- and sgRNA/ActRNA:t-adapted uPEn editing respectively into proximal and distal positions. **(A)** The pegRNAs were designed to edit at the distal positions of several sites (see Figure 1). PE7/pegRNAs were transfected into HEK293T cells. The targeted sites were subjected to NGS analyses (n = 3 biological replicates, mean±SD). The accurate edits, as well as the imprecise prime edits and the indels from both groups are presented. (**B, C**) A target location in *IL2RB* was chosen based on its closer proximity to the downstream, than the upstream

cleavable positions. The pegRNA (B) and the sgRNA/ActRNA:t (C), respectively corresponding to editing upon the downstream and upstream cleavage sites, were constructed. HEK293T cells were transfected with uPEn/pegRNA or uPEn/sgRNA/ActRNA:t. The targeted sites were subjected to NGS analyses. For each editing condition, the representative allele frequency graphs are displayed. The downward arrow represents the cleavage position, and the PAM sequence is highlighted by a black box. The accurate alleles are marked by a hand sign. The major prime edits (accurate and imprecise) and the indel-type edits are displayed separately, as indicated. All alleles observed with frequency ≥ 0.50% are displayed.

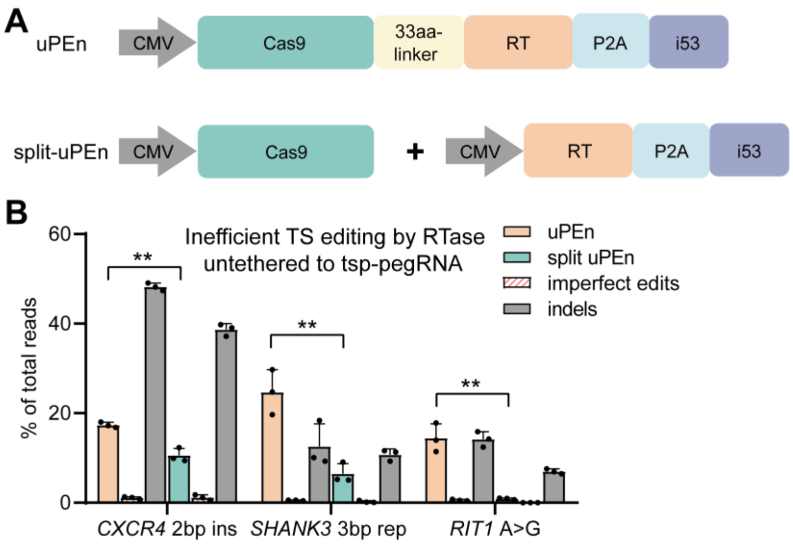

Supplementary Fig. S3: The efficiencies of uPEn or the RT-separated split uPEn in tsp-pegRNA-dependent editing. (**A**) The schematic drawing of uPEn or the RT-separated split uPEn are shown. (**B**) HEK293T cells were subjected to editing (into proximal positions) by tsp-pegRNA-adapted uPEn or split uPEn at three genomic loci. The targeted sites were subjected to NGS analyses (n = 3 biological replicates, mean±SD). The accurate edits, as well as the imprecise prime edits and the indels from both groups are presented. The differences in the levels of accurate edits between uPEn and the split uPEn groups were determined using two-tailed Student's *t*-test (**: $P < 0.01$). The actual P values are 0.0021, 0.0046 and 0.0019 for *CXCR4*, *SHANK3* and *RIT1* sites, respectively.

A

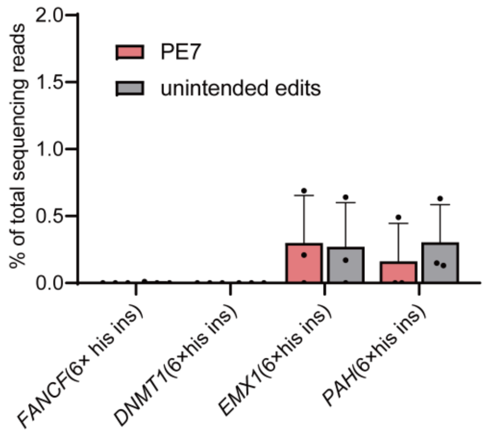

Supplementary Fig. S4: Low efficiencies by PE7 for installation of insertions at distal positions. The efficiencies by PE7/pegRNAs for 4 loci (the same sites in Fig. 3E) were determined (HEK293T cells, n = 3 biological replications).

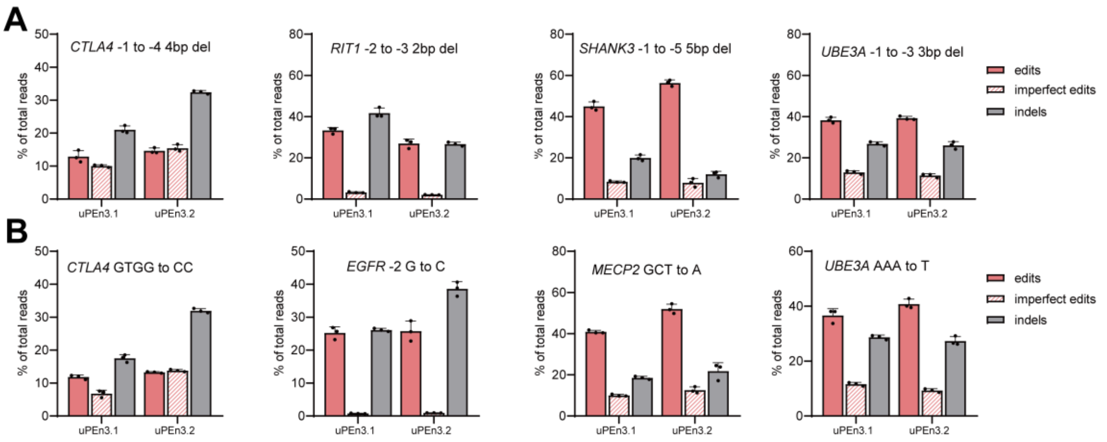

Supplementary Fig. S5: The uPEn3.1 and uPEn3.2 perform comparably for small deletion and replacement edits. Evaluation of uPEn3.1 and uPEn3.2 for various small deletional (A, 4 loci) and substitutional edits (B, 4 loci) in HEK293T cells. The targeted sites were subjected to NGS analyses (n = 3 biological replications, mean±SD). The accurate edits, as well as the imprecise prime edits and the indels from both groups are presented.

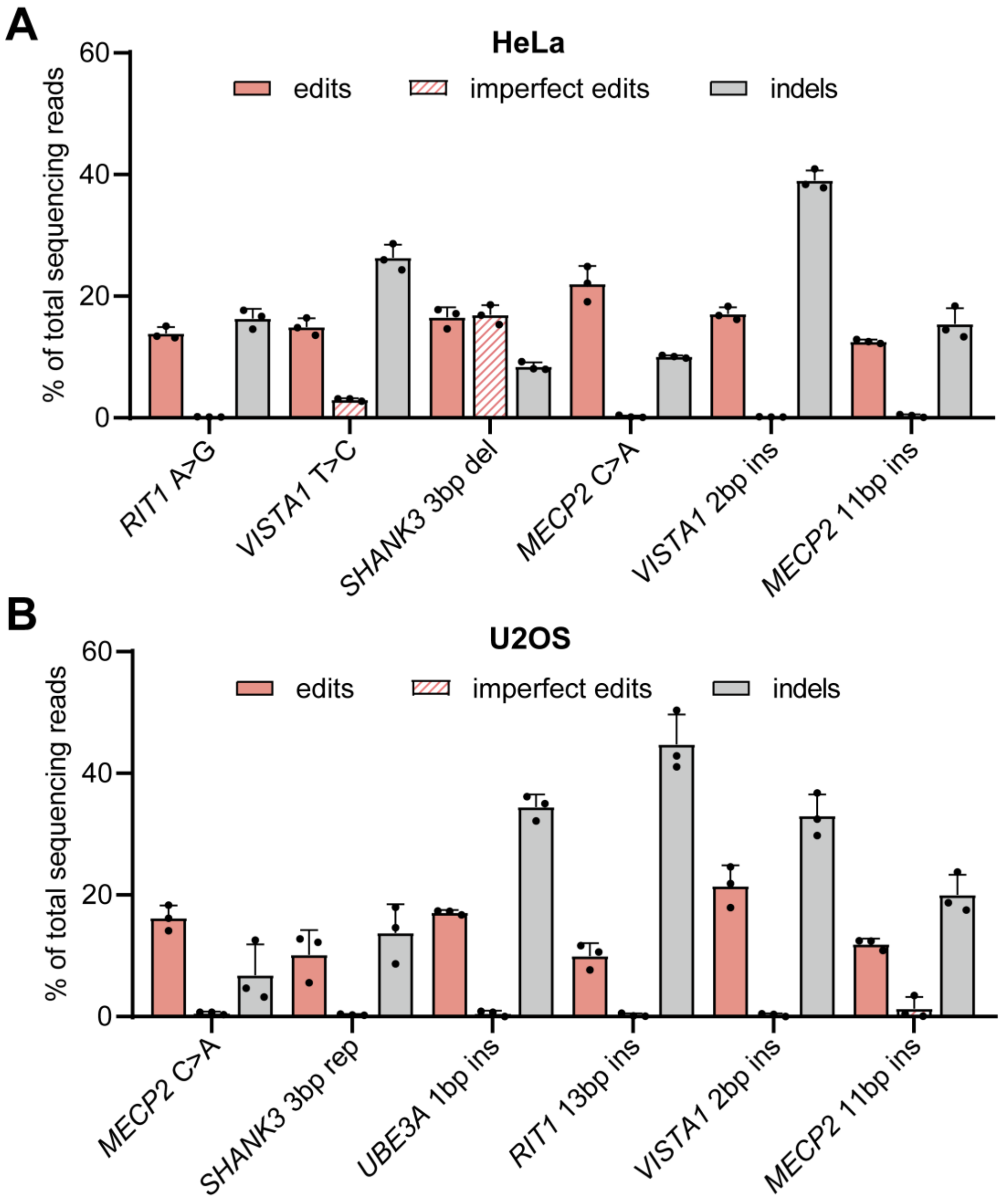

Supplementary Fig. S6: Examination of uPEn/tsp-pegRNA (uPEn3.2) editing in other human cell lines. (**A, B**) The uPEn3.2 was applied for different types of edits into proximal positions in HeLa (A) and U2OS cells (B). The targeted sites were subjected to NGS analyses (n = 3 biological replicates, mean±SD). The accurate edits, as well as the imprecise prime edits and the indels from both groups are presented.

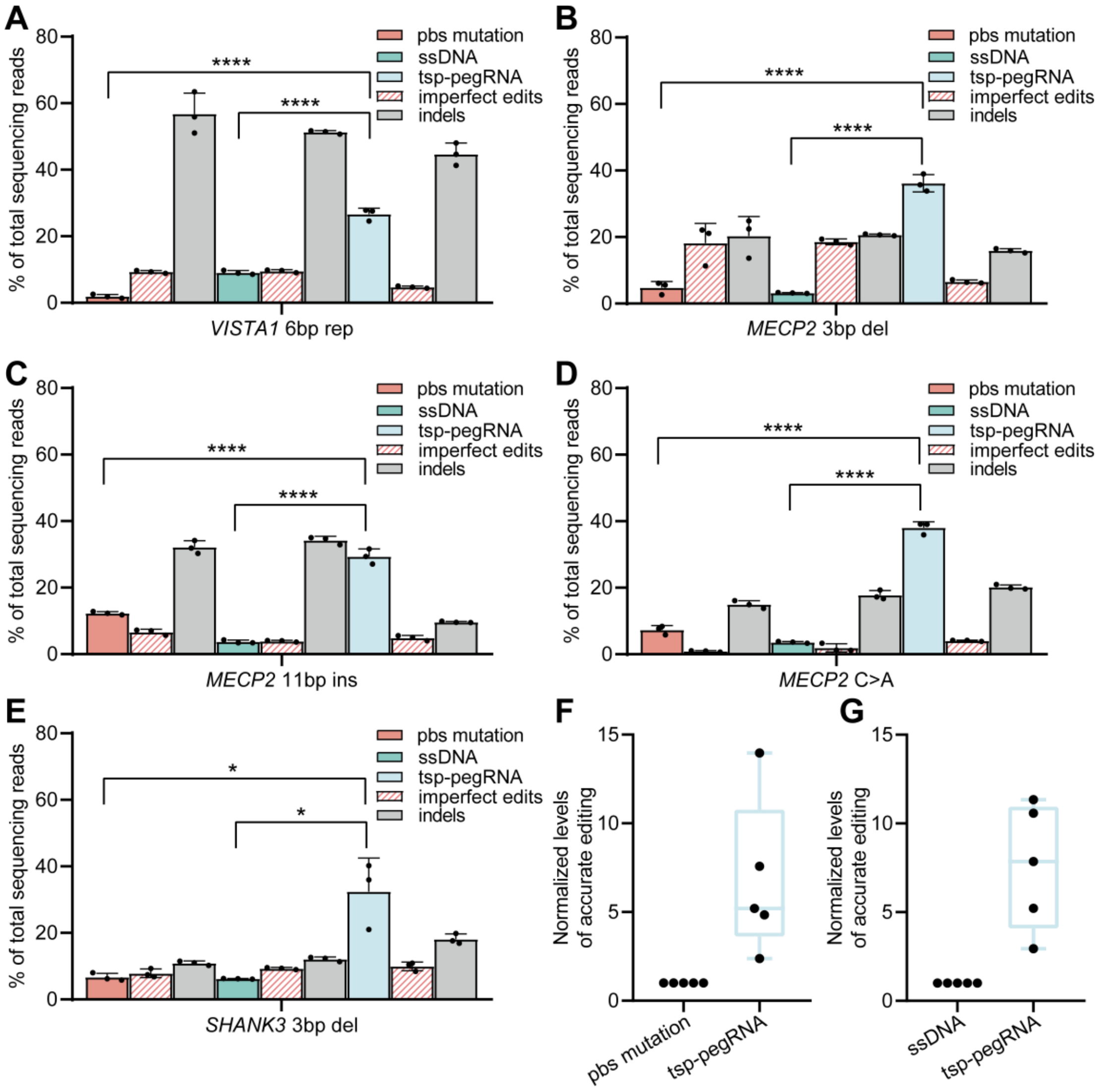

Supplementary Fig. S7: Comparisons of uPEn/tsp-pegRNA (uPEn3.2) editing with other TS-editing options. (**A-E**) The conventional Cas9/HDR method could adopt ssDNA complementary to the TS strand as template for programmed editing [ssDNA group]. Furthermore, it is possible that an alternative uPEn NTS-editing strategy based on a variant pegRNA bearing the small edits in the PBS portion (as opposite to the usual placements in the RTT region) might also enable edits at the upstream side (relative to the PAM) of DSB [PBS mutation group]. For the conventional HDR platform, ssDNA templates bearing the intended modifications, and with 35-bp homology arms complementary to the TS sequences were adopted. HEK293T cells were transfection with constructs corresponding to the PBS mutation group, ssDNA group and the uPEn/tsp-pegRNA group. Five different genomic loci were tested for the

same modifications that correspond to proximal-position editing for tsp-pegRNA. The targeted sites were subjected to NGS analyses (n = 3 biological replicates, mean±SD). The accurate edits, as well as the imprecise prime edits and the indels from three groups are presented. The differences in the levels of accurate edits between the pegRNA and the two TS-editing groups were determined using two-tailed Student's *t*-test (*: $P < 0.05$, ****: $P < 0.0001$). (**F, G**) The results for editing at all sites were summarized. In (F), the efficiencies of tsp-pegRNA-dependent editing is normalized per site to the levels of the PBS mutation group. In (G), the efficiencies of tsp-pegRNA-dependent editing is normalized per site to the levels of the ssDNA group. Results are presented in box plots. The center line shows medians of all data points (n = 5 genomic loci) and the box limits correspond to the upper the lower quartiles, while the whiskers extend to the largest and smallest values.

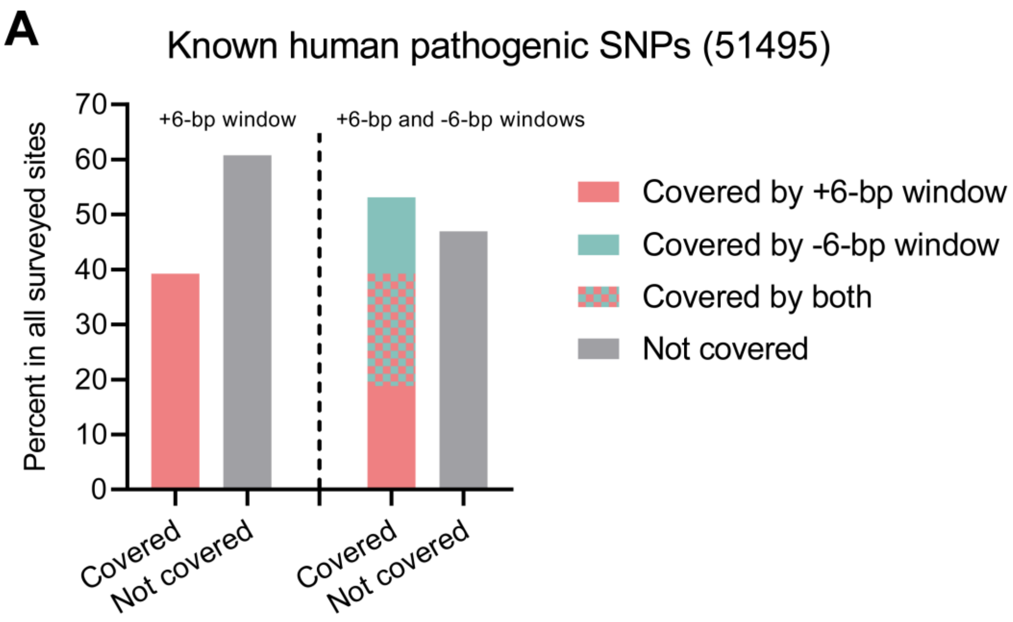

Supplementary Fig. S8: Enablement of TS editing could significantly expand the targetable space of prime editors. We specified positions within 6 bp (+1 to +6) downstream of the nCas9 (Cas9) cleavage point as "favorable editing window" for PE and standard uPEn. Given that uPEn3.1/3.2 mediate upstream-directed editing, they could additionally cover the proximal positions between -1 and -6 relative to the cleavage point ("-6-bp window"). Subsequently, we surveyed the coverage of ClinVar-documented human pathogenic SNPs by the +6-bp window and -6-bp window.

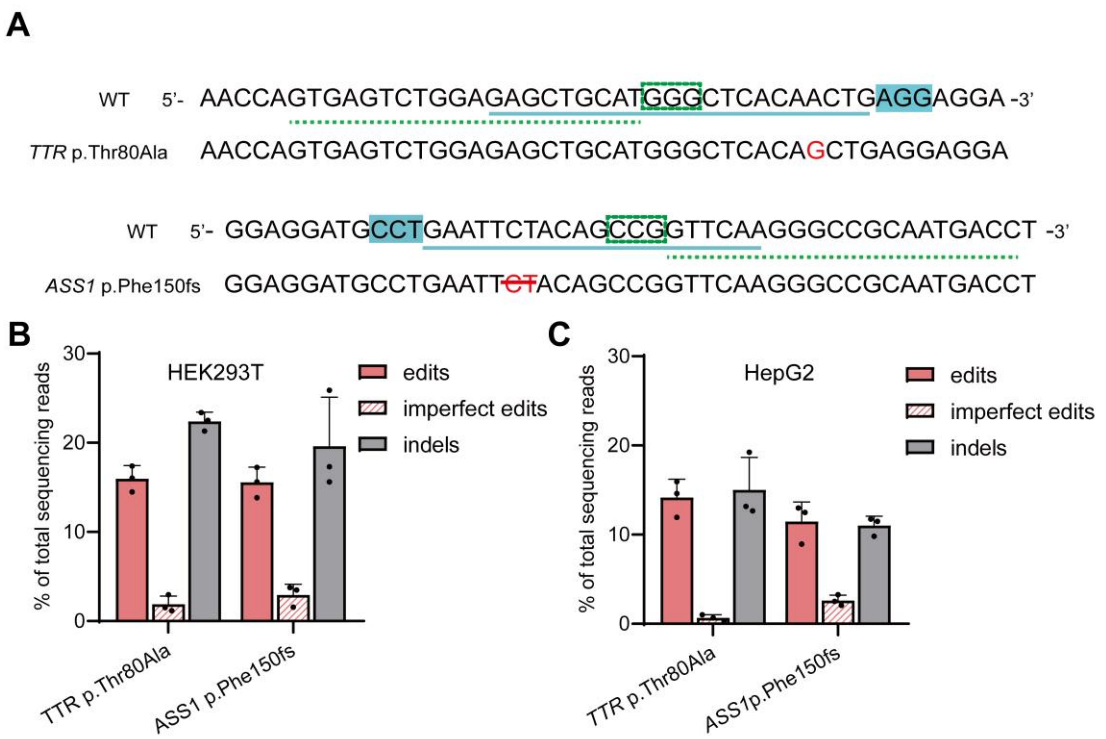

Supplementary Fig. S9: Application of uPEn3.2 for installation of two pathogenic mutations in HEK293T cells. (**A**) The gene segments around the pathogenic mutations in *TTR* and ASS1 are illustrated. The altered bases in comparison to the WT sequences are marked in red. Moreover, the PAM (or its complementary motif) locations corresponding to the downstream cleavable positions are marked by blue shades, while the 20-bp target sites are underlined (blue). On the other hand, the PAM (or its complementary motif) locations corresponding to the upstream cleavable positions are marked by green boxes with dotted line, while the 20-bp target sites are underlined (green dotted line). The locations of the intended mutations are apparently more adjacent to the downstream cleavable position, than the upstream cleavable position. (**B, C**) Therefore, tsp-pegRNAs were designed to direct uPEn (uPEn3.2) to install these mutations in HEK293T cells (B) and HepG2 cells (C). The targeted sites were subjected to NGS analyses (n = 3 biological replicates, mean±SD). The accurate edits, as well as the imprecise prime edits and the indels are presented.

A

FANCF	Target sequence	PAM	Control	sgRNA/ActRNA:t	tsp-pegRNA
On-target	GGAATCCCTTCTGCAGCACC	TGG		41.11	29.84
Off-target1	GGAAcCCCgTCTGCAGCACC	AGG	0.01	3.29	4.54
Off-target2	tGAATCCCaTCTcCAGCACC	AGG	0.01	0.01	0.01
Off-target3	GGAATCCCTggTGCAGCttC	AGG	0.01	0.01	0.01
EMX1					
On-target	CGGCTTTACCATAGAGTCCT	TGG		54.10	39.69
Off-target1	aGGCTTTACCATAGAGgCtT	CGG	0.32	0.31	0.35
Off-target2	CGGCTTTgCCATAcAGTtCT	CGG	0.01	0.01	0.01
Off-target3	CtGgTTTcCCATAGAGcCCT	AGG	0.16	0.24	0.24
PAH					
On-target	TGGTTTCCGCCTCCGACCTG	TGG		75.79	69.29
Off-target1	TGcTTTCtGCCTCaGACCTc	AGG	0.01	0.01	0.01
Off-target2	cGGTTTCCGCCTCCGAgCTG	GGG	0.01	0.20	0.01
Off-target3	TGGTTTCCcCaTCaGACCTG	AGG	0.01	0.01	0.01

Supplementary Fig. S10: Off-target analyses for uPEn3.1~3.2. Some potential off-target (OT) sites corresponding to the guide RNA sequences of sgRNAs/tsp-pegRNAs (for uPEn3.1/uPEn3.2) against three target sites (within *FANCF*, *EMX1* and *PAH*) were overall randomly selected from CasOFFinder-nominated lists. One exception was the OT1-*FANCF*, which was a previously established true OT site. The control groups were set to be transfections with uPEn and canonical pegRNAs whose spacer sequences were independent of those for sgRNA and tsp-pegRNA. Indeed, these pegRNAs were designed to target the immediate upstream cleavable sites in relation to the sgRNA and tsp-pegRNA targets. Given the differences in spacer sequences, the pegRNA groups constituted proper controls for the respective sgRNA and tsp-pegRNA groups. HEK293T cells were transfected with the control, uPEn3.1 or uPEn3.2 against different target sites (for 6x His tag insertion). The target and OT sites were subsequently analyzed by NGS (n = 3 biological replicates, [except for the *PAH*-tsp-pegRNA group with 2 replicates]). The mean levels of sequence modifications (%) at each site in the uPEn3.1 and 3.2 groups were compared with those in the control group. The expected activities of control pegRNAs (independent of uPEn3.1 or uPEn3.2's target sequences) were confirmed by the rates of their on-target edits (31.5%, 30.3% and 44.73% for the *FANCF*, *EMX1* and *PAH* groups, respectively).

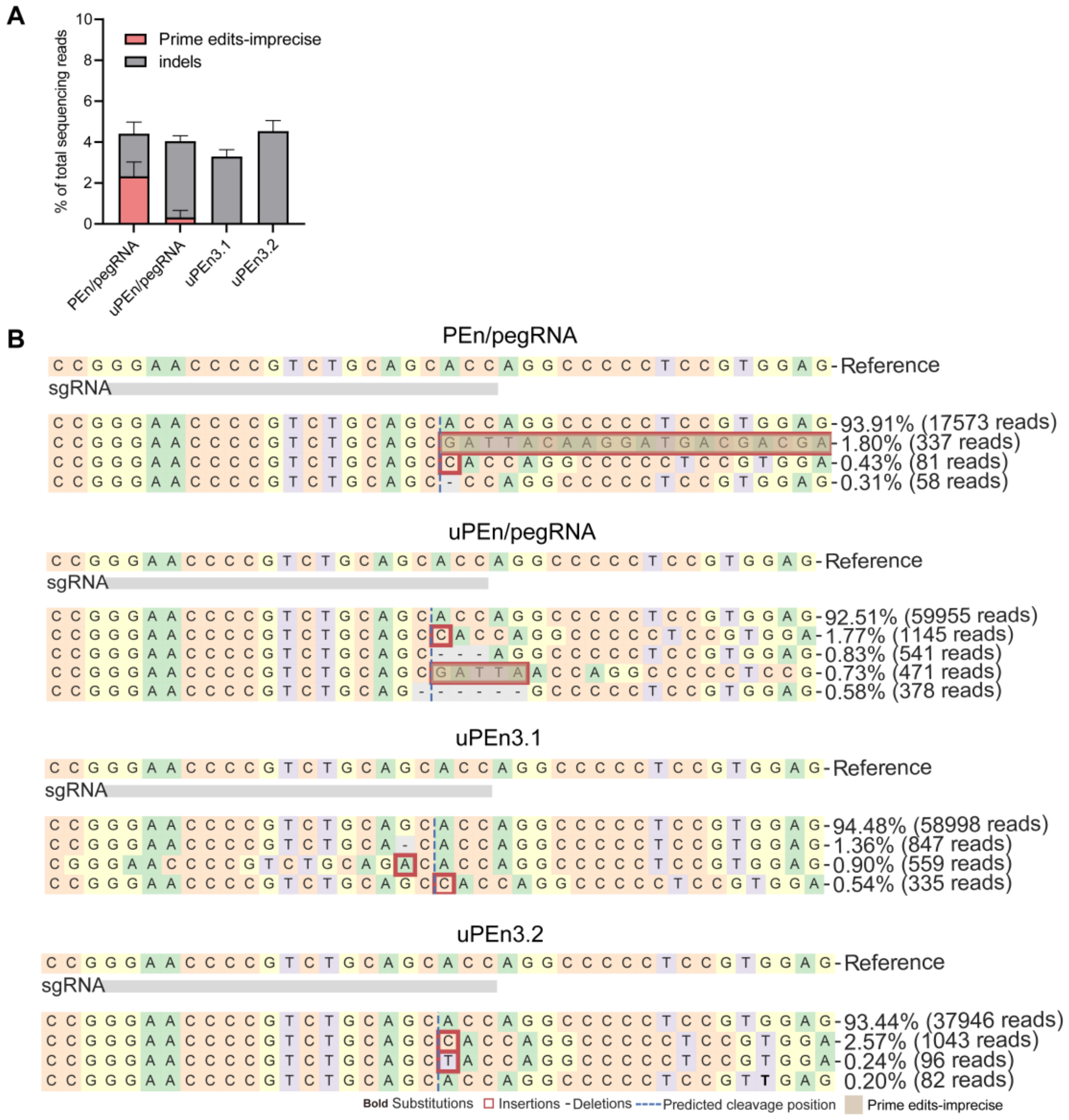

Supplementary Fig. S11: Analyses of potential off-target priming events by standard uPEn, uPEn3.1 and uPEn3.2. We utilized NGS data from our previous work [uploaded at NCBI-SRA: RJNA847383] corresponding to PEn (uPEn) hits at the reference OT1 site of pegRNA-*FANCF*. In addition, the current NGS data at the same off-target site (OT1-*FANCF* for both uPEn3.1/3.2, Supplementary Fig. S10) were analyzed. (**A**) The various allele types shown by CRISPResso were quantitated. Some alleles indicative of RT-dependent edits at the off-target site could be identified in the standard (u)PEn groups. (**B**) The representative allele frequency graphs corresponding to respective nuclease PEs are displayed.

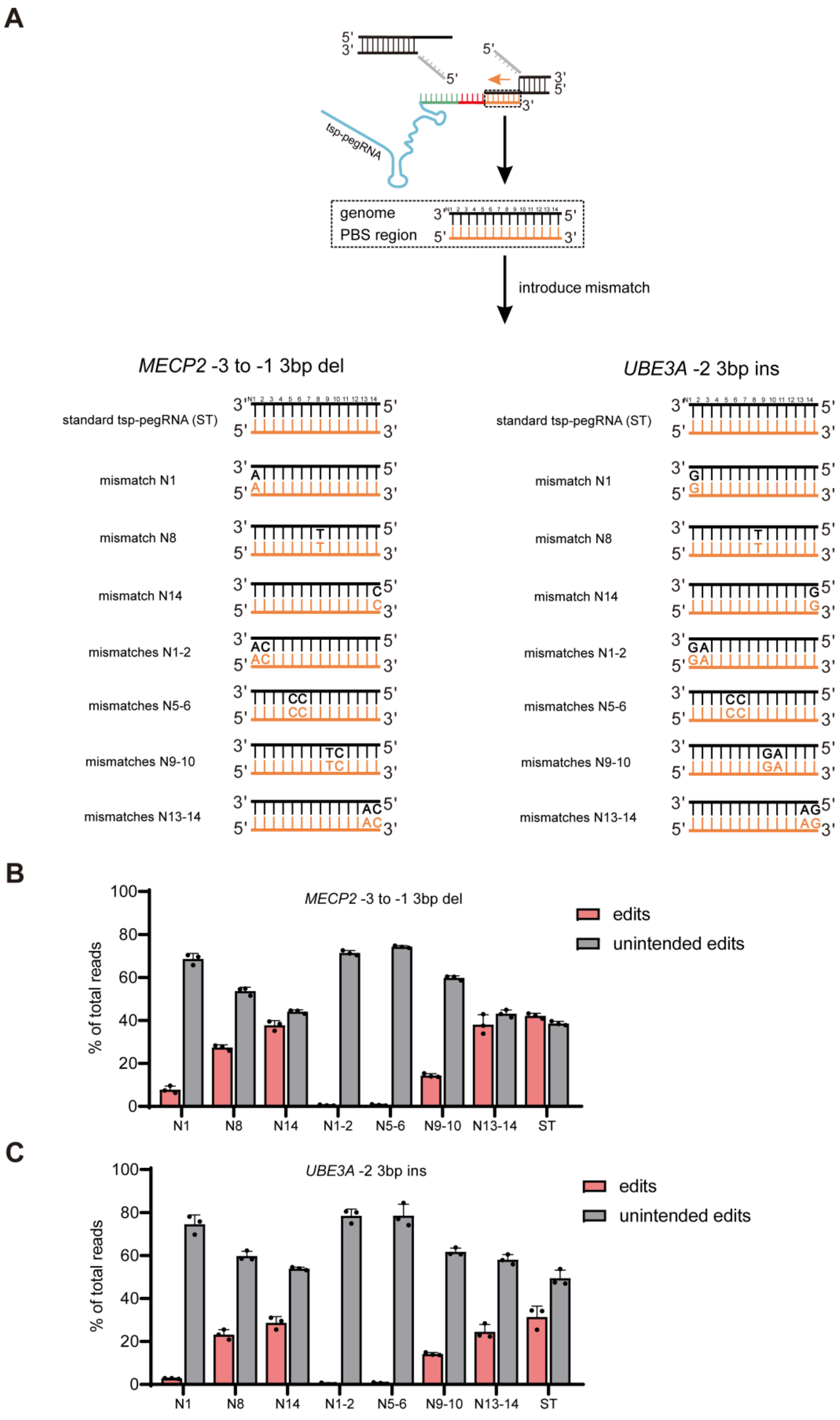

Supplementary Fig. S12: uPEn3.2-dependent prime editing requires significant

homologies between the PBS of tsp-pegRNA and the DSB end. (**A**) The illustrations show the positions of mismatches introduced to the PBS of two groups of tsp-pegRNAs. (**B, C**) HEK293T cells were transfected with uPEn3.2-guided by the tsp-pegRNAs designed as above. The targeted sites were subjected to NGS analyses. Rates of correct edits and unintended edits are presented (n = 3 biological replicates, mean±SD).

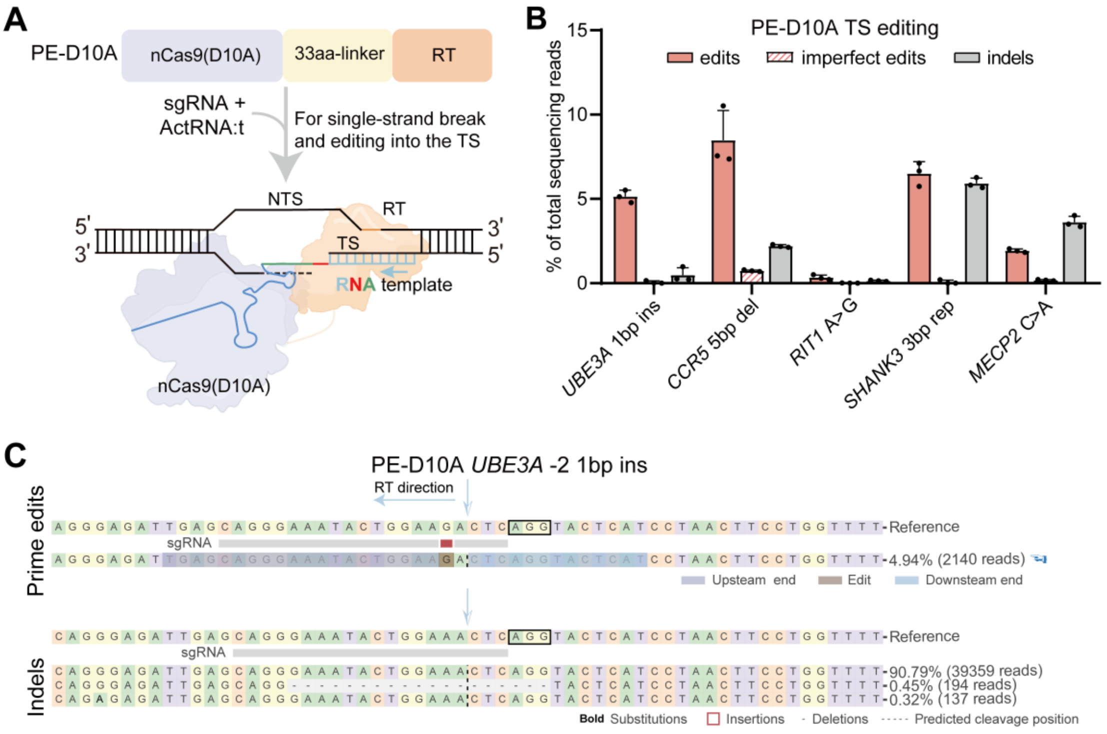

Supplementary Fig. S13: Low levels of TS editing by sgRNA/ActRNA:t-adapted nickase PE (D10A). (**A**) Potential editing framework for TS-editing nickase PE (D10A) is depicted. The variant PE (D10A) would first nick the TS. If a mechanism similar to that of uPEn TS editing would possibly apply to the nickase system, a correspondingly designed ActRNA:t could potentially also direct TS editing by the PE (D10A). (**B**) The targeting sgRNAs and the corresponding ActRNA:t-s were constructed to program proximal edits (mostly at -2 positions) at various genomic loci. HEK293T cells were subjected to editing with sgRNA/ActRNA:t-adapted PE-D10A. The targeted sites were subjected to NGS analyses (n = 3 biological replicates, mean±SD). The accurate edits, as well as the imprecise prime edits and the indels from both groups are presented. (**C**) The representative allele frequency graphs for editing at the *UBE3A* site are presented. The downward arrow represents the cleavage position, and the PAM sequence is highlighted by a black box. The accurate alleles are marked by a hand sign. The prime edit and the indel-type edits are displayed separately, as indicated. All alleles observed with frequency ≥ 0.20% are displayed.

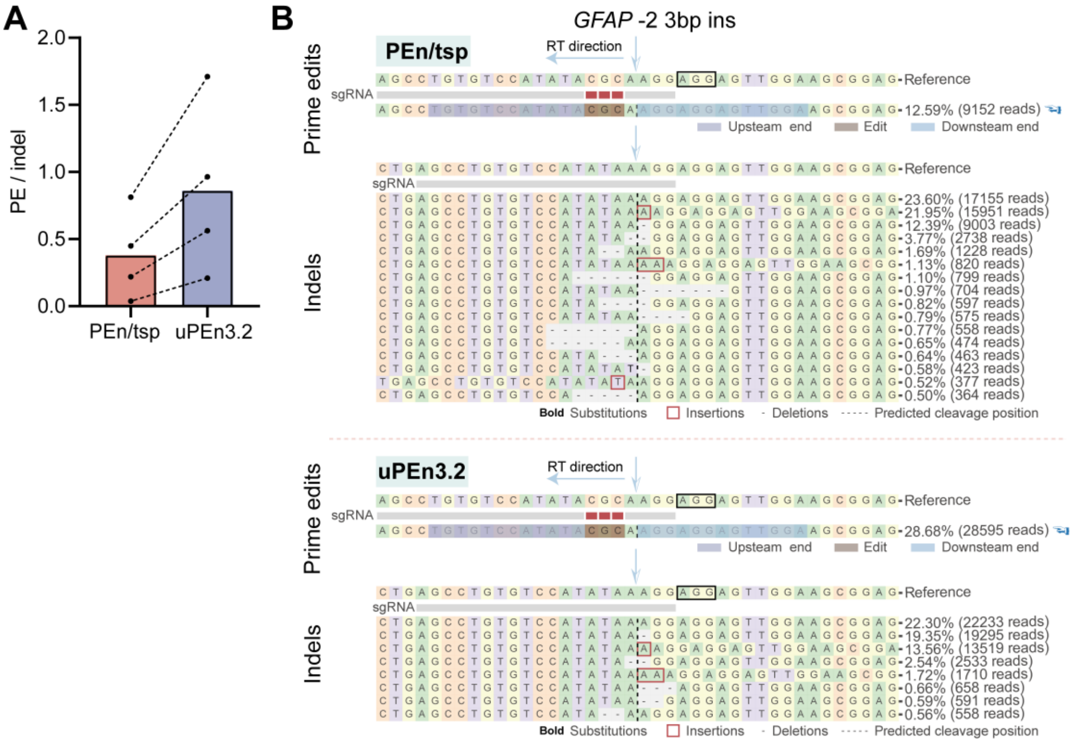

Supplementary Fig. S14: The role of the i53 module in tsp-pegRNA-directed uPEn editing. (**A**) The effects of tsp-pegRNAs in combination with uPEn (with i53, uPEn3.2) and PEn (without i53, PEn/tsp) were compared for programming proximal edits at 4 genomic loci. The accurate edits, as well as the imprecise prime edits and the indels from both groups were determined (see Fig. 4E). The rates of accurate edits and indels showed significant, reciprocal changes. The ratios of accurate edits/indels for each group are further presented here. The bar graphs show the average difference (n = 4 genomic sites). The dotted lines connect data points at the same site. (**B**) The representative allele frequency graphs for editing at the *GFAP* site are presented. The downward arrow represents the cleavage position, and the PAM sequence is highlighted by a black box. The accurate alleles are marked by a hand sign. The major prime edits and the indel-type edits are displayed separately, as indicated. All alleles observed with frequency ≥ 0.50% are displayed.

A

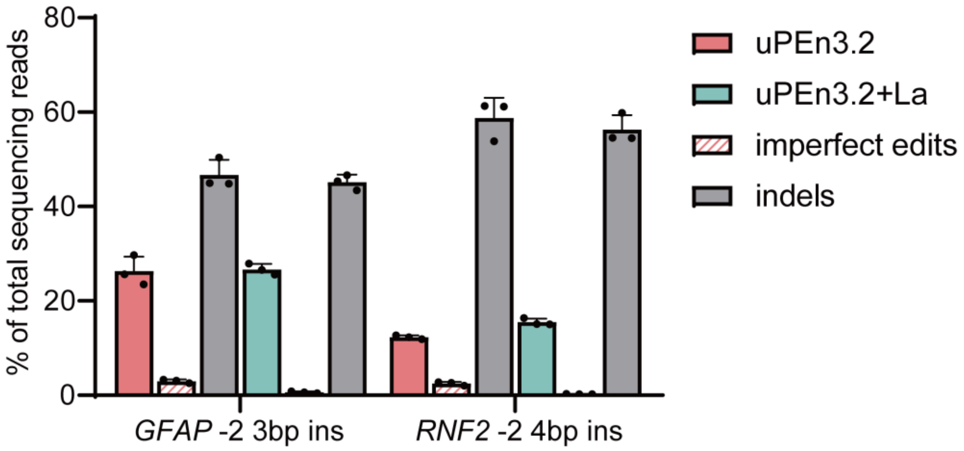

Supplementary Fig. S15: The use of La protein module together with uPEn3.2 did not lead to enhanced performances. The La protein module (1-194) that stabilizes the 3′ end of Pol III-dependent small RNAs was adopted. For targeting two sites shown in Fig. 4E, HEK293T cells were transfected with uPEn3.2 components together with the La protein module. The targeted sites were subjected to NGS analyses. The accurate edits, as well as the imprecise prime edits and the indels are presented (n = 3 biological replicates, mean±SD). The quantitative results for the control groups with uPEn/tsp-pegRNA-only are the same as in Fig. 4E.

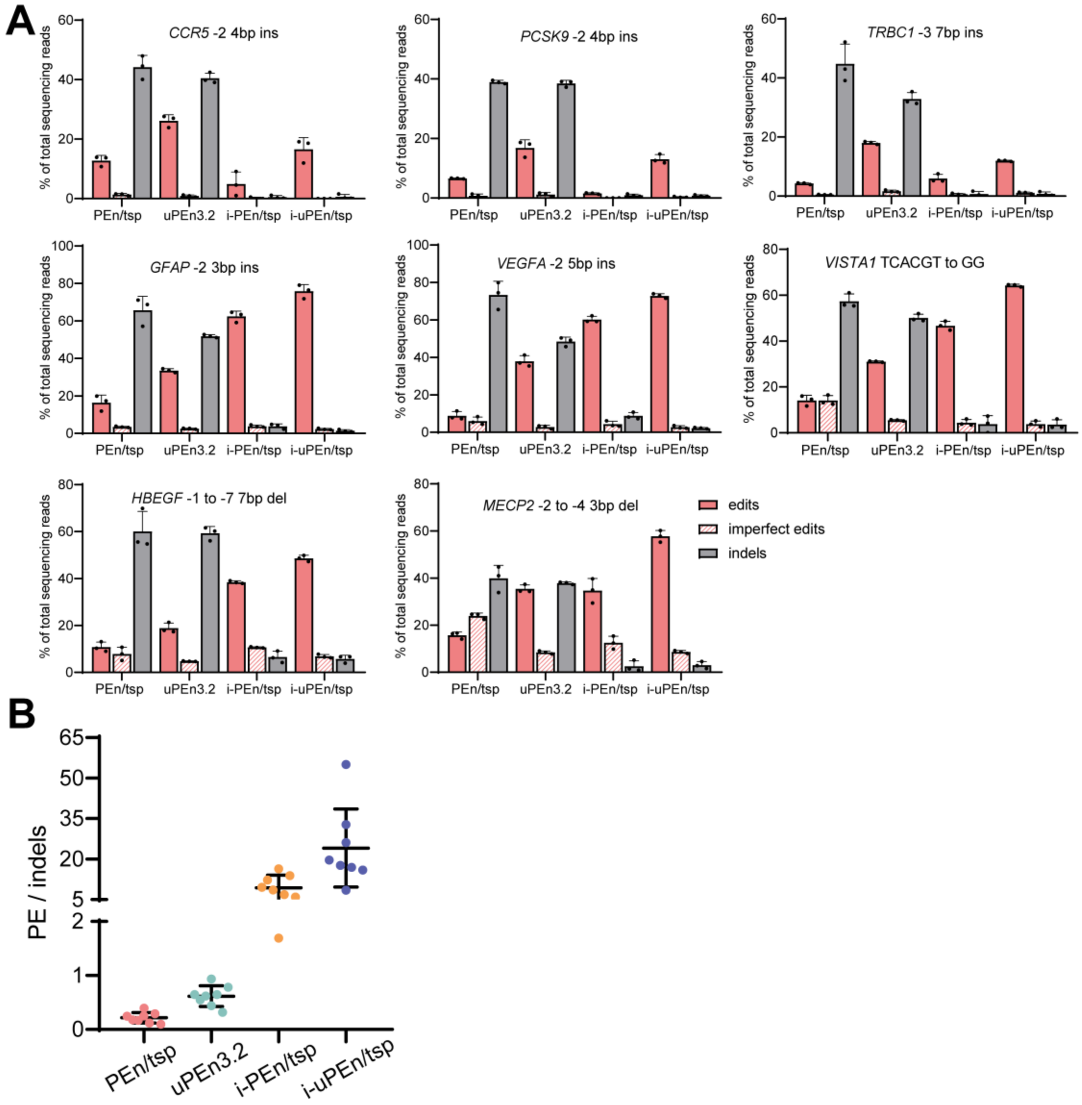

Supplementary Fig. S16: Administration of AZD7648 with tsp-pegRNA-adapted PEn and uPEn led to robust inhibition of editing impurities. HEK293T cells were pretreated with or without 1 μM of AZD7648 for 3 h and were subsequently transfected with tsp-pegRNA-adapted PEn or uPEn (for 8 different sites). (**A**) The targeted sites were subjected to NGS analyses. The tsp-pegRNA-guided PEn, uPEn, and their AZD7648-coaddition counterparts are respectively marked as PEn/tsp, uPEn3.2, i-PEn/tsp and i-uPEn/tsp, respectively. The accurate edits, as well as the imprecise prime edits and the indels are presented (n = 3 biological replicates, mean±SD). (**B**) The ratios of accurate edits/indels for each site edited by the respective editors are further summarized here. The mean values and standard deviations from the respective editor groups are presented.

A

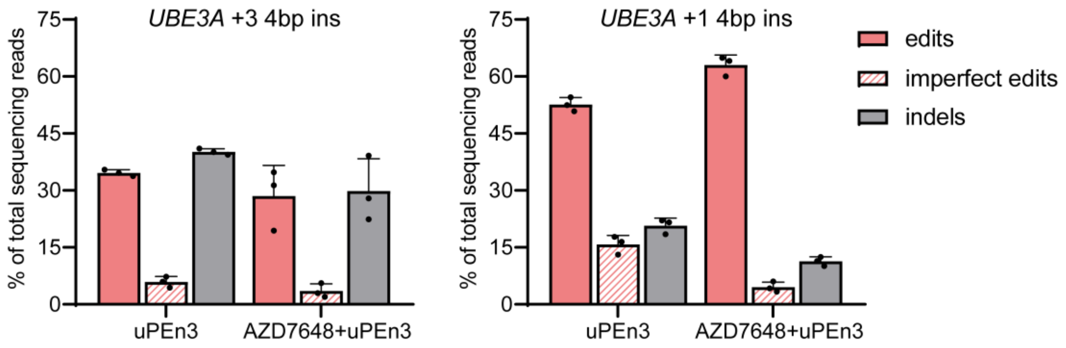

Supplementary Fig. S17: The indel rates by standard uPEn were relatively refractory to AZD7648 co-administration. HEK293T cells were pretreated with or without 1 μM of AZD7648 for 3 h and were subsequently transfected with standard uPEn3 (for 2 different edits). The targeted sites were subjected to NGS analyses. The accurate edits, as well as the imprecise prime edits and the indels are presented (n = 3 biological replicates, mean±SD).

A

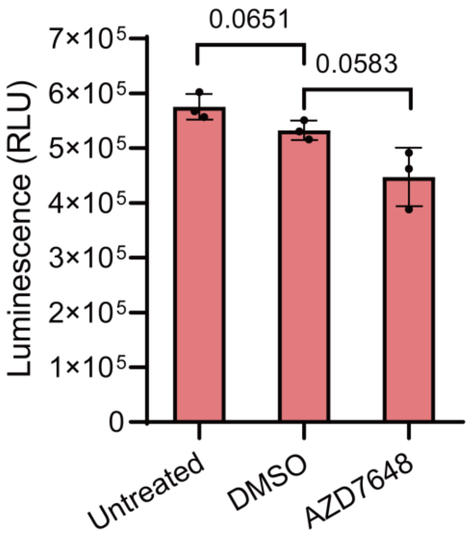

Supplementary Fig. S18: The effects of AZD7648 treatment on cell viability. HEK293T cells were seeded at 5×10^3 cells/well in 96-well plates. 24 h after seeding, cells were treated with ±1 μM AZD7648. 72 h after the treatment, cell viability was determined with CellTiter-Lumi Plus Luminescence Cell Viability Assay kit according to the manufacturer’s protocol (n = 3 biological replicates, mean±SD).

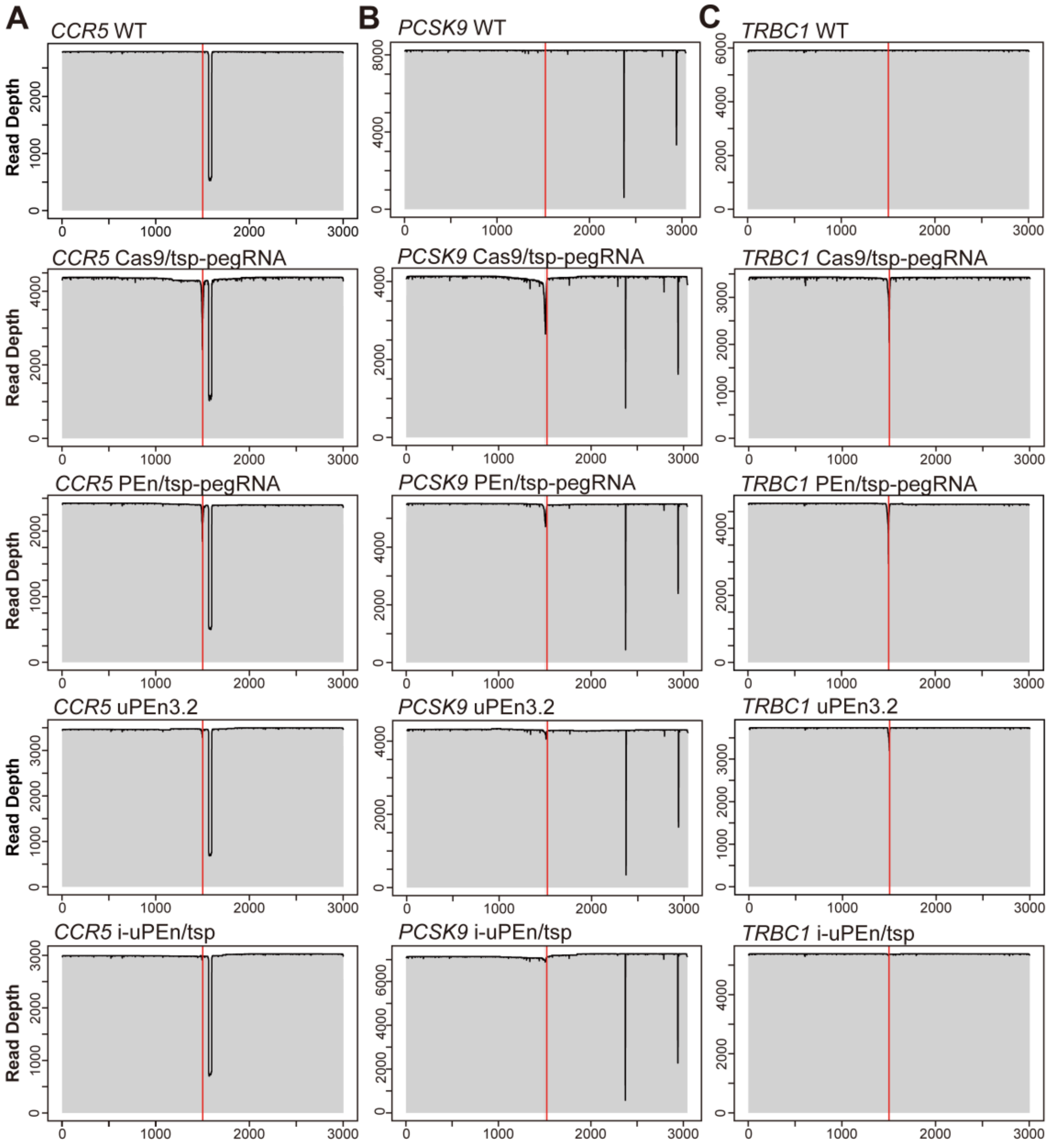

Supplementary Fig. S19: Assessment of on-target large deletions induced by tsp-pegRNA-guided Cas9, PEn and uPEn, as well as by i-uPEn/tsp. HEK293T cells were edited at *CCR5* (**A**), *PCSK9* (**B**), and *TRBC1* (**C**) loci with tsp-pegRNA-guided Cas9, PEn and uPEn, as well as by i-uPEn/tsp for 72 h. The unedited cells were used as a negative control (WT). The targeted sites were subjected to PacBio long-read sequencing analyses.

Supplementary Tables

Supplementary Table 1. Primers used for constructing of various types of pegRNA and sgRNA plasmids.

Backbone pegRNA-F	TTTTTTTAAAGAATTCTCGACCTCGAGACA
Backbone pegRNA-R	CGGTGTTTCGTCCTTTCCACA

Supplementary Table 2. Primers used for ssDNA donors

VISTA1-rep-ssDNA	GCAGACCTGCTCAGAGCGCCGGCCCATCCGCAACCGGTCCAGGACCTTCACCTGCACCATGGAGGCCACCAG
MECP2-del-ssDNA	TGGACACGGAAGCTTAAGCAAAGGAAATCTGGCCGTGCTGGGAAGTATGATGTGTATTTGATCAAGTAAG
MECP2-ins-ssDNA	GGACACGGAAGCTTAAGCAAAGGAAATCTGGCCGCAACGTCAGGAATCTGCTGGGAAGTATGATGTGTATTTGATCAAGTAAG
MECP2-sub-ssDNA	GACACGGAAGCTTAAGCAAAGGAAATCTGGCCGCTATGCTGGGAAGTATGATGTGTATTTGATCAAGTAAG
SHANK3-del-ssDNA	GCCAGCCTTCCCGGCGCTGCAGTATCTCGAGTCGGACGTGGAGGGTGTGGCCTGGAGGGCCGGGCTGCGC

Supplementary Notes

Supplementary Note 1. The sequence of PEn.

NLS

Cas9 nuclease

Linker

Reverse transcriptase

ATGaaacggacagccgacggaagcgagttcgagtcaccaaagaagaagcggaaagtcgacaagaagtacagcatc
ggcctggacatcggcaccaactctgtgggctgggccgtgatcaccgacgagtacaaggtgcccagcaagaaattcaagg
tgctgggcaacaccgaccggcacagcatcaagaagaacctgatcggagccctgctgttcgacagcggcgaaacagccg
aggccacccggctgaagagaaccgccagaagaagatacaccagacggaagaaccggatctgctatctgcaagagatc
ttcagcaacgagatggccaaggtggacgacagcttcttccacagactggaagagtccttcctggtggaagaggataagaa
gcacgagcggcaccccatcttcggcaacatcgtggacgaggtggcctaccacgagaagtaccccaccatctaccacctg
agaaagaaactggtggacagcaccgacaaggccgacctgcggctgatctatctggccctggcccacatgatcaagttccg
gggccacttcctgatcgagggcgacctgaaccccgacaacagcgacgtggacaagctgttcatccagctggtgcagacct
acaaccagctgttcgaggaaaaccccatcaacgccagcggcgtggacgccaaggccatcctgtctgccagactgagca
agagcagaaagctggaaaatctgatcgcccagctgcccggcgagaagaagaatggcctgttcggaaacctgattgccct
gagcctgggcctgacccccaacttcaagagcaacttcgacctggccgaggatgccaaactgcagctgagcaaggacac
ctacgacgacgacctggacaacctgctggcccagatcggcgaccagtacgccgacctgtttctggccgccaagaacctgt
ccgacgccatcctgctgagcgacatcctgagagtgaacaccgagatcaccaaggcccccctgagcgcctctatgatcaag
agatacgacgagcaccaccaggacctgaccctgctgaaagctctcgtgcggcagcagctgcctgagaagtacaaagag
attttcttcgaccagagcaagaacggctacgccggctacattgacggcggagccagccaggaagagttctacaagttcatc
aagcccatcctggaaaagatggacggcaccgaggaactgctcgtgaagctgaagagagaggacctgctgcggaagca
gcggaccttcgacaacggcagcatcccccaccagatccacctgggagagctgcacgccattctgcggcggcaggaaga
tttttacccattcctgaaggacaaccgggaaaagatcgagaagatcctgaccttccgcatcccctactacgtgggccctctgg
ccaggggaaacagcagattcgcctggatgaccagaaagagcgaggaaaccatcaccccctggaacttcgaggaagtg
gtggacaagggcgcttccgcccagagcttcatcgagcggatgaccaacttcgataagaacctgcccaacgagaaggtgc
tgcccaagcacagcctgctgtacgagtacttcaccgtgtataacgagctgaccaaagtgaaatacgtgaccgagggaatg
agaaagcccgccttcctgagcggcgagcagaaaaaggccatcgtggacctgctgttcaagaccaaccggaaagtgacc
gtgaagcagctgaaagaggactacttcaagaaaatcgagtgcttcgactccgtggaaatctccggcgtggaagatcggttc
aacgcctccctgggcacataccacgatctgctgaaaattatcaaggacaaggacttcctggacaatgaggaaaacgagg
acattctggaagatatcgtgctgaccctgacactgtttgaggacagagagatgatcgaggaacggctgaaaacctatgccc
acctgttcgacgacaaagtgatgaagcagctgaagcggcggagatacaccggctggggcaggctgagccggaagctg
atcaacggcatccgggacaagcagtccggcaagacaatcctggatttcctgaagtccgacggcttcgccaacagaaactt
catgcagctgatccacgacgacagcctgacctttaaagaggacatccagaaagcccaggtgtccggccagggcgatagc
ctgcacgagcacattgccaatctggccggcagccccgccattaagaagggcatcctgcagacagtgaaggtggtggacg
agctcgtgaaagtgatgggccggcacaagcccgagaacatcgtgatcgaaatggccagagagaaccagaccacccag
aagggacagaagaacagccgcgagagaatgaagcggatcgaagagggcatcaaagagctgggcagccagatcctg
aaagaacaccccgtggaaaacacccagctgcagaacgagaagctgtacctgtactacctgcagaatgggcgggatatgt
acgtggaccaggaactggacatcaaccggctgtccgactacgatgtggacCAtatcgtgcctcagagctttctgaaggac
gactccatcgacaacaaggtgctgaccagaagcgacaagaaccggggcaagagcgacaacgtgccctccgaagagg
tcgtgaagaagatgaagaactactggcggcagctgctgaacgccaagctgattacccagagaaagttcgacaatctgacc
aaggccgagagaggcggcctgagcgaactggataaggccggcttcatcaagagacagctggtggaaacccggcagat
cacaaagcacgtggcacagatcctggactcccggatgaacactaagtacgacgagaatgacaagctgatccgggaagt

gaaagtgatcaccctgaagtccaagctggtgtccgatttccggaaggatttccagttttacaaagtgcgcgagatcaacact
accaccacgcccacgacgcctacctgaacgccgtcgtgggaaccgccctgatcaaaaagtaccctaagctggaaagcg
agttcgtgtacggcgactacaaggtgtacgacgtgcggaagatgatcgccaagagcgagcaggaaatcggcaaggcta
ccgccaagtacttcttctacagcaacatcatgaactttttcaagaccgagattaccctggccaacggcgagatccggaagcg
gcctctgatcgagacaaacggcgaaaccggggagatcgtgtgggataagggccgggattttgccaccgtgcggaaagtg
ctgagcatgccccaagtgaatatcgtgaaaaagaccgaggtgcagacaggcggcttcagcaaagagtctatcctgccca
agaggaacagcgataagctgatcgccagaaagaaggactgggaccctaagaagtacggcggcttcgacagccccacc
gtggcctattctgtgctggtggtggccaaagtggaaaagggcaagtccaagaaactgaagagtgtgaaagagctgctggg
gatcaccatcatggaaagaagcagcttcgagaagaatcccatcgactttctggaagccaagggctacaaagaagtgaaa
aaggacctgatcatcaagctgcctaagtactccctgttcgagctggaaaacggccggaagagaatgctggcctctgccgg
cgaactgcagaagggaaacgaactggccctgccctccaaatatgtgaacttcctgtacctggccagccactatgagaagct
gaagggctcccccgaggataatgagcagaaacagctgtttgtggaacagcacaagcactacctggacgagatcatcgag
cagatcagcgagttctccaagagagtgatcctggccgacgctaatctggacaaagtgctgtccgcctacaacaagcaccg
ggataagcccatcagagagcaggccgagaatatcatccacctgtttaccctgaccaatctgggagcccctgccgccttcaa
gtactttgacaccaccatcgaccggaagaggtacaccagcaccaaagaggtgctggacgccaccctgatccaccagag
catcaccggcctgtacgagacacggatcgacctgtctcagctgggaggtgactccggcggaagctctggtggcagcaagc
ggaccgccgacggctctgaattcgagagccctaagaagaaaagaaaggtgagcggaggctctagcggcggaagcacc
ctgaacattgaagacgagtatagactgcatgaaacaagcaaggaacccgacgtgtccctgggctccacctggctgtccga
ctttccccaggcctgggccgagacaggaggaatgggcctggccgtgcggcaggcacccctgatcatccctctgaaggcc
acctctacacccgtgagcatcaagcagtaccctatgtctcaggaggccagactgggcatcaagcctcacatccagaggct
gctggaccagggcatcctggtgccatgccagagcccctggaacacaccactgctgcccgtgaagaagccaggcaccaa
tgactatagacccgtgcaggatctgagagaggtgaacaagagggtggaggatatccaccccaccgtgcccaacccttac
aatctgctgtccggcctgcccccttctcaccagtggtatacagtgctggacctgaaggatgccttcttttgtctgagactgcacc
ctaccagccagccactgttcgcctttgagtggagggaccctgagatgggcatctctggccagctgacctggacacgcctgc
ctcagggcttcaagaatagcccaacactgtttaacgaggccctgcaccgcgacctggcagatttccggatccagcaccca
gatctgatcctgctgcagtacgtggacgatctgctgctggccgccaccagcgagctggattgccagcagggaacacgcgc
cctgctgcagaccctgggaaacctgggatatagggcatccgccaagaaggcccagatctgtcagaagcaggtgaagtac
ctgggctatctgctgaaggagggccagagatggctgacagaggccaggaaggagacagtgatgggccagccaacacc
caagaccccaagacagctgagggagttcctgggcaaagcaggatttgcaggctgttcatcccaggattcgcagagatgg
cagcacctctgtacccactgaccaagccgggcaccctgtttaattggggccctgaccagcagaaggcctatcaggagatc
aagcaggccctgctgacagcaccagccctgggcctgccagacctgaccaagcctttcgagctgtttgtggatgagaagca
gggctacgccaagggcgtgctgacccagaagctgggaccatggagacggcccgtggcctatctgtccaagaagctgga
cccagtggcagcaggatggccaccatgcctgaggatggtggcagcaatcgccgtgctgacaaaggatgccggcaagct
gaccatgggacagccactggtcatcctggcaccacacgcagtggaggccctggtgaagcagcctccagatcgctggctgt
ctaacgcccggatgacacactaccaggccctgctgctggacaccgatcgcgtgcagtttggccctgtggtggccctgaatcc
agccaccctgctgcctctgccagaggagggcctgcagcacaactgtctggacatcctggcagaggcacacggaacaag
gccagacctgaccgatcagcccctgcctgacgccgatcacacatggtataccgatggaagctccctgctgcaggagggcc
agaggaaggcaggagcagcagtgaccacagagacagaagtgatctgggccaaggccctgccagcaggcacatccgc
ccagcgggccgagctgatcgccctgacccaggccctgaagatggccgagggcaagaagctgaacgtgtacacagactc
cagatatgccttcgccaccgcacacatccacggagagatctacaggcgccggggctggctgacctctgagggcaaggag
atcaagaacaaggatgagatcctggccctgctgaaggccctgtttctgcccaagcggctgagcatcatccactgtcctggac
accagaagggacactccgccgaggcaaggggcaatcggatggccgaccaggccgccagaaaggctgctattactgaa
actcccgacacttccactctgctgattgaaaactcctccccttctggcggctcaaaaagaaccgccgacggcagcgaattcg
agtctcccaagaagaagaggaaagtcggctctggccctgccgctaagagagtgaagctggactaa

Supplementary Note 2. The sequence of uPEn.

NLS

Cas9 nuclease

Linker

Reverse transcriptase

P2A

Ubiquitin

ATGaaacggacagccgacggaagcgagttcgagtcaccaaagaagaagcggaaagtcgacaagaagtacagcatc
ggcctggacatcggcaccaactctgtgggctgggccgtgatcaccgacgagtacaaggtgcccagcaagaaattcaagg
tgctgggcaacaccgaccggcacagcatcaagaagaacctgatcggagccctgctgttcgacagcggcgaaacagccg
aggccacccggctgaagagaaccgccagaagaagatacaccagacggaagaaccggatctgctatctgcaagagatc
ttcagcaacgagatggccaaggtggacgacagcttcttccacagactggaagagtccttcctggtggaagaggataagaa
gcacgagcggcaccccatcttcggcaacatcgtggacgaggtggcctaccacgagaagtaccccaccatctaccacctg
agaaagaaactggtggacagcaccgacaaggccgacctgcggctgatctatctggccctggcccacatgatcaagttccg
gggccacttcctgatcgagggcgacctgaaccccgacaacagcgacgtggacaagctgttcatccagctggtgcagacct
acaaccagctgttcgaggaaaaccccatcaacgccagcggcgtggacgccaaggccatcctgtctgccagactgagca
agagcagaaagctggaaaatctgatcgcccagctgcccggcgagaagaagaatggcctgttcggaaacctgattgccct
gagcctgggcctgacccccaacttcaagagcaacttcgacctggccgaggatgccaaactgcagctgagcaaggacac
ctacgacgacgacctggacaacctgctggcccagatcggcgaccagtacgccgacctgtttctggccgccaagaacctgt
ccgacgccatcctgctgagcgacatcctgagagtgaacaccgagatcaccaaggcccccctgagcgcctctatgatcaag
agatacgacgagcaccaccaggacctgaccctgctgaaagctctcgtgcggcagcagctgcctgagaagtacaaagag
attttcttcgaccagagcaagaacggctacgccggctacattgacggcggagccagccaggaagagttctacaagttcatc
aagcccatcctggaaaagatggacggcaccgaggaactgctcgtgaagctgaagagagaggacctgctgcggaagca
gcggaccttcgacaacggcagcatcccccaccagatccacctgggagagctgcacgccattctgcggcggcaggaaga
tttttacccattcctgaaggacaaccgggaaaagatcgagaagatcctgaccttccgcatcccctactacgtgggccctctgg
ccaggggaaacagcagattcgcctggatgaccagaaagagcgaggaaaccatcaccccctggaacttcgaggaagtg
gtggacaagggcgcttccgcccagagcttcatcgagcggatgaccaacttcgataagaacctgcccaacgagaaggtgc
tgcccaagcacagcctgctgtacgagtacttcaccgtgtataacgagctgaccaaagtgaaatacgtgaccgagggaatg
agaaagcccgccttcctgagcggcgagcagaaaaaggccatcgtggacctgctgttcaagaccaaccggaaagtgacc
gtgaagcagctgaaagaggactacttcaagaaaatcgagtgcttcgactccgtggaaatctccggcgtggaagatcggttc
aacgcctccctgggcacataccacgatctgctgaaaattatcaaggacaaggacttcctggacaatgaggaaaacgagg
acattctggaagatatcgtgctgaccctgacactgtttgaggacagagagatgatcgaggaacggctgaaaacctatgccc
acctgttcgacgacaaagtgatgaagcagctgaagcggcggagatacaccggctggggcaggctgagccggaagctg
atcaacggcatccgggacaagcagtccggcaagacaatcctggatttcctgaagtccgacggcttcgccaacagaaactt
catgcagctgatccacgacgacagcctgacctttaaagaggacatccagaaagcccaggtgtccggccagggcgatagc
ctgcacgagcacattgccaatctggccggcagccccgccattaagaagggcatcctgcagacagtgaaggtggtggacg
agctcgtgaaagtgatgggccggcacaagcccgagaacatcgtgatcgaaatggccagagagaaccagaccacccag
aagggacagaagaacagccgcgagagaatgaagcggatcgaagagggcatcaaagagctgggcagccagatcctg
aaagaacaccccgtggaaaacacccagctgcagaacgagaagctgtacctgtactacctgcagaatgggcgggatatgt
acgtggaccaggaactggacatcaaccggctgtccgactacgatgtggacCAtatcgtgcctcagagctttctgaaggac
gactccatcgacaacaaggtgctgaccagaagcgacaagaaccggggcaagagcgacaacgtgccctccgaagagg
tcgtgaagaagatgaagaactactggcggcagctgctgaacgccaagctgattacccagagaaagttcgacaatctgacc
aaggccgagagaggcggcctgagcgaactggataaggccggcttcatcaagagacagctggtggaaacccggcagat
cacaaagcacgtggcacagatcctggactcccggatgaacactaagtacgacgagaatgacaagctgatccgggaagt

gaaagtgatcaccctgaagtccaagctggtgtccgatttccggaaggatttccagttttacaaagtgcgcgagatcaacaact
accaccacgcccacgacgcctacctgaacgccgtcgtgggaaccgccctgatcaaaaagtaccctaagctggaaagcg
agttcgtgtacggcgactacaaggtgtacgacgtgcggaagatgatcgccaagagcgagcaggaaatcggcaaggcta
ccgccaagtacttcttctacagcaacatcatgaactttttcaagaccgagattaccctggccaacggcgagatccggaagcg
gcctctgatcgagacaaacggcgaaaccggggagatcgtgtgggataagggccgggattttgccaccgtgcggaaagtg
ctgagcatgccccaagtgaatatcgtgaaaaagaccgaggtgcagacaggcggcttcagcaaagagtctatcctgccca
agaggaacagcgataagctgatcgccagaaagaaggactgggaccctaagaagtacggcggcttcgacagccccacc
gtggcctattctgtgctggtggtggccaaagtggaaaagggcaagtccaagaaactgaagagtgtgaaagagctgctggg
gatcaccatcatggaaagaagcagcttcgagaagaatcccatcgactttctggaagccaagggctacaaagaagtgaaa
aaggacctgatcatcaagctgcctaagtactccctgttcgagctggaaaacggccggaagagaatgctggcctctgccgg
cgaactgcagaagggaaacgaactggccctgccctccaaatatgtgaacttcctgtacctggccagccactatgagaagct
gaagggctcccccgaggataatgagcagaaacagctgtttgtggaacagcacaagcactacctggacgagatcatcgag
cagatcagcgagttctccaagagagtgatcctggccgacgctaatctggacaaagtgctgtccgcctacaacaagcaccg
ggataagcccatcagagagcaggccgagaatatcatccacctgtttaccctgaccaatctgggagcccctgccgccttcaa
gtactttgacaccaccatcgaccggaagaggtacaccagcaccaaagaggtgctggacgccaccctgatccaccagag
catcaccggcctgtacgagacacggatcgacctgtctcagctgggaggtgactccggcggaagctctggtggcagcaagc
ggaccgccgacggctctgaattcgagagccctaagaagaaaagaaaggtgagcggaggctctagcggcggaagcacc
ctgaacattgaagacgagtatagactgcatgaaacaagcaaggaacccgacgtgtccctgggctccacctggctgtccga
ctttccccaggcctgggccgagacaggaggaatgggcctggccgtgcggcaggcacccctgatcatccctctgaaggcc
acctctacacccgtgagcatcaagcagtaccctatgtctcaggaggccagactgggcatcaagcctcacatccagaggct
gctggaccagggcatcctggtgccatgccagagcccctggaacacaccactgctgcccgtgaagaagccaggcaccaa
tgactatagacccgtgcaggatctgagagaggtgaacaagagggtggaggatatccaccccaccgtgcccaacccttac
aatctgctgtccggcctgccccctctcaccagtggtatacagtgctggacctgaaggatgccttcttttgtctgagactgcacc
ctaccagccagccactgttcgcctttgagtggagggaccctgagatgggcatctctggccagctgacctggacacgcctgc
ctcagggcttcaagaatagcccaacactgtttaacgaggccctgcaccgcgacctggcagatttccggatccagcaccca
gatctgatcctgctgcagtacgtggacgatctgctgctggccgccaccagcgagctggattgccagcagggaacacgcgc
cctgctgcagaccctgggaaacctgggatatagggcatccgccaagaaggcccagatctgtcagaagcaggtgaagtac
ctgggctatctgctgaaggagggccagagatggctgacagaggccaggaaggagacagtgatgggccagccaacacc
caagaccccaagacagctgagggagttcctgggcaaagcaggattttgcaggctgttcatcccaggattcgcagagatgg
cagcacctctgtacccactgaccaagccgggcaccctgtttaattggggccctgaccagcagaaggcctatcaggagatc
aagcaggccctgctgacagcaccagccctgggcctgccagacctgaccaagcctttcgagctgtttgtggatgagaagca
gggctacgccaagggcgtgctgacccagaagctgggaccatggagacggcccgtggcctatctgtccaagaagctgga
cccagtggcagcaggatggccaccatgcctgaggatggtggcagcaatcgccgtgctgacaaaggatgccggcaagct
gaccatgggacagccactggtcatcctggcaccacacgcagtggaggccctggtgaagcagcctccagatcgctggctgt
ctaacgcccggatgacacactaccaggccctgctgctggacaccgatcgcgtgcagtttggccctgtggtggccctgaatcc
agccaccctgctgcctctgccagaggagggcctgcagcacaactgtctggacatcctggcagaggcacacggaacaag
gccagacctgaccgatcagcccctgcctgacgccgatcacacatggtataccgatggaagctccctgctgcaggagggcc
agaggaaggcaggagcagcagtgaccacagagacagaagtgatctgggccaaggccctgccagcaggcacatccgc
ccagcgggccgagctgatcgccctgacccaggccctgaagatggccgagggcaagaagctgaacgtgtacacagactc
cagatatgccttcgccaccgcacacatccacggagagatctacaggcgccggggctggctgacctctgagggcaaggag
atcaagaacaaggatgagatcctggccctgctgaaggccctgtttctgcccaagcggctgagcatcatccactgtcctggac
accagaagggacactccgccgaggcaaggggcaatcggatggccgaccaggccgccagaaaggctgctattactgaa
actcccgacacttccactctgctgattgaaaactcctccccttctggcggctcaaaaagaaccgccgacggcagcgaattcg
agtctcccaagaagaagaggaaagtcggctctggccctgccgctaagagagtgaagctggacggatccggcgcaacaa

acttctctctgctgaaacaagccggagatgtcgaagagaatcctggaccgatgcatatgaaacggacagccgacggaag
cgagttcgagtcaccaaagaagaagcggaaagtcgccgccagtttaaacggcgcgccattaattaaggatccaatgttga
ttttcgtgaaaacccttaccgggaaaaccatcaccctcgaggttgaaccctcggatacgatagaaaatgtaaaggccaaga
tccaggataaggaaggaattcctcctgatcagcagagactggcctttgctggcaaatcgctggaagatggacgtactttgtct
gactacaatattctaaaggactctaaacttcatcctctgttgagacttcgttaa
